# Pushing the limits of HiFi assemblies reveals centromere diversity between two *Arabidopsis thaliana* genomes

**DOI:** 10.1093/nar/gkac1115

**Published:** 2022-12-01

**Authors:** Fernando A Rabanal, Maike Gräff, Christa Lanz, Katrin Fritschi, Victor Llaca, Michelle Lang, Pablo Carbonell-Bejerano, Ian Henderson, Detlef Weigel

**Affiliations:** Department of Molecular Biology, Max Planck Institute for Biology Tübingen, 72076 Tübingen, Germany; Department of Molecular Biology, Max Planck Institute for Biology Tübingen, 72076 Tübingen, Germany; Department of Molecular Biology, Max Planck Institute for Biology Tübingen, 72076 Tübingen, Germany; Department of Molecular Biology, Max Planck Institute for Biology Tübingen, 72076 Tübingen, Germany; Genomics Technologies, Corteva Agriscience, Johnston, IA 50131, USA; Genomics Technologies, Corteva Agriscience, Johnston, IA 50131, USA; Department of Molecular Biology, Max Planck Institute for Biology Tübingen, 72076 Tübingen, Germany; Department of Plant Sciences, University of Cambridge, Cambridge, CB2 3EA, UK; Department of Molecular Biology, Max Planck Institute for Biology Tübingen, 72076 Tübingen, Germany

## Abstract

Although long-read sequencing can often enable chromosome-level reconstruction of genomes, it is still unclear how one can routinely obtain gapless assemblies. In the model plant *Arabidopsis thaliana*, other than the reference accession Col-0, all other accessions *de novo* assembled with long-reads until now have used PacBio continuous long reads (CLR). Although these assemblies sometimes achieved chromosome-arm level contigs, they inevitably broke near the centromeres, excluding megabases of DNA from analysis in pan-genome projects. Since PacBio high-fidelity (HiFi) reads circumvent the high error rate of CLR technologies, albeit at the expense of read length, we compared a CLR assembly of accession Eyach15-2 to HiFi assemblies of the same sample. The use of five different assemblers starting from subsampled data allowed us to evaluate the impact of coverage and read length. We found that centromeres and rDNA clusters are responsible for 71% of contig breaks in the CLR scaffolds, while relatively short stretches of GA/TC repeats are at the core of >85% of the unfilled gaps in our best HiFi assemblies. Since the HiFi technology consistently enabled us to reconstruct gapless centromeres and 5S rDNA clusters, we demonstrate the value of the approach by comparing these previously inaccessible regions of the genome between the Eyach15-2 accession and the reference accession Col-0.

## INTRODUCTION

The first reference genome of *Arabidopsis thaliana*, from the accession Columbia (Col-0), was completed in the year 2000 with Sanger sequencing and assembled by a BAC minimal tiling path approach (1). It has served for over two decades as the gold standard because the chromosome arms were assembled to very high quality, with several minor improvements made after the initial release (2). While extremely useful for analysis of euchromatic genes, the original *A. thaliana* genome assembly only poorly represented the most repetitive fraction of the genome, centromeres and ribosomal RNA gene clusters. The genomes of several other accessions were subsequently assembled based on Illumina paired-end reads, but either consisting of thousands of scaffolds or containing reference sequences in regions that were difficult to assemble (3–5). Recently, the contiguity of *de novo* assemblies has been greatly improved with long-read sequencing, such as Oxford Nanopore Technologies (ONT) (reviewed in (6,7)) and PacBio single-molecule real-time (SMRT) in the original continuous long read (CLR) sequencing mode (8), despite both of them having relatively high per-base error rates of individual sequencing reads. To date, the genomes of 16 *A. thaliana* accessions sequenced with PacBio CLR technology have been published (9–18). These assemblies commonly achieved several chromosome arm-level contigs, but they invariably stopped short of assembling through centromeric and pericentromeric regions as well as rDNA clusters. Only last year has the first gapless centromere assembly been published for the *A. thaliana* reference accession Col-0, primarily from ultra-long ONT reads complemented by PacBio high-fidelity (HiFi) reads for gap closing and polishing (19). Paradoxically, and despite rice (*Oryza sativa*) and maize (*Zea mays*) having much larger genomes than *A. thaliana*, PacBio CLR technology has been successfully exploited to assemble gapless centromeres for about a third of the chromosomes in pan-genome analyses of 31 rice (20) and 26 maize accessions (21,22). This likely reflects fundamental differences in the composition of their centromeres. For instance, the tandem satellite repeats CentC (∼156 bp long) in maize are confined to a few small blocks interspersed with numerous centromeric retrotransposons (23). In contrast, the tandem *CEN180* satellite repeats (∼178 bp long) in *A. thaliana* Col-0 form very large arrays, only interrupted by 111 interspersed sequences larger than 1 kb (19).

PacBio HiFi reads, which are >99% accurate because they are generated from circular consensus sequencing (24), overcome the high error limitation of ONT and CLR technologies at the cost of reducing read length. Recent studies in humans, rice and barley that compared HiFi-based assemblies to other long-read technologies showed mostly an enhanced correctness, completeness and—sometimes—an improved contiguity (25–29). Those three metrics are often referred to as the ‘three C’s’ and provide important information about the assembly quality. Among the most commonly used HiFi assemblers, both FALCON (12) and Canu (30) were originally conceived for PacBio CLR data. However, since the emergence of PacBio HiFi reads, a HiFi-optimized parameter became available in FALCON (24), while HiCanu emerged as a modification of the original Canu assembler (31). In contrast, Hifiasm (32), Peregrine (33) and IPA (github.com/PacificBiosciences/pbipa) were developed specifically for the purpose of assembling HiFi data.

Here, we compared genome assemblies of the *A. thaliana* accession Eyach15-2 based on a single CLR library with assemblies based on a single HiFi library and processed with five different state-of-the-art assemblers. We evaluated the impact of both coverage and read length in the metrics of contiguity, completeness and correctness, for which we analyzed 280 HiFi assemblies based on subsets of the original HiFi data. We paid particular attention to the repetitive fraction of the genome and explored in detail the likely causes of contig breaks between both PacBio technologies and the different HiFi assemblers. Since the HiFi technology enabled us to obtain gapless centromeric regions, we present the first comparison of these previously unassembled regions of the genome between two *A. thaliana* accessions.

## MATERIALS AND METHODS

### Plant growth conditions

*Arabidopsis thaliana* seeds of the natural strains Eyach15-2 (Ey15-2; 1001 Genomes Project accession ID 9994; North American Arabidopsis Stock Center ID CS76399) and Columbia-0 (Col-0; 1001 Genomes Project accession ID 6909; North American Arabidopsis Stock Center ID CS76778) were germinated on soil and stratified in darkness at 4°C for 6 days, after which they were transferred to long-day conditions (16 h light) at 23°C and 65% relative humidity under 110–140 μmol m^−2^ s^−1^ light provided by GreenPower TLED modules (Philips Lighting GmbH, Hamburg, Germany). To reduce starch accumulation, 21-day-old and 26-day-old plants of Ey15-2 and Col-0, respectively, were placed into darkness for 24 h before harvesting. For Ey15-2, ca. 30 g of flash-frozen rosettes from multiple individuals were ground in liquid nitrogen with pestle and mortar. For Col-0, a single individual was harvested and processed in a similar manner.

### High molecular weight DNA extraction

For Ey15-2, we extracted high molecular weight DNA (HMW-DNA) as described (17). Briefly, tissue powder was resuspended in 500 ml of freshly prepared, ice-cold nuclei isolation buffer (NIB: 10 mM Tris pH 8, 100 mM KCl, 10 mM EDTA pH 8, 500 mM sucrose, 4 mM spermidine, 1 mM spermine). The homogenate was filtered through two layers of miracloth (EMD Millipore; #475855-1R) and distributed into several 50 ml Falcon tubes, to which 1:20 (v/v) of NIB containing 20% Triton-X-100 was added. Samples were incubated on ice for 15 min, and centrifuged at 3000 g at 4°C for 15 min. Nuclei pellets were pooled, washed with approximately 35 ml of NIB containing 1% Triton-X-100, and further centrifuged at 3000 g at 4°C for 15 min. The pellet was gently resuspended in 20 ml of pre-warmed (37°C) G2 lysis buffer (Qiagen; Cat. no. 1014636), incubated with 50 μg/ml RNaseA (Qiagen; #19101) at 37°C for 30 min, followed by 200 μg/ml proteinase K treatment (Qiagen; #19133) at 50°C for 3 h. After centrifugation at 8000 g at 4°C for 15 min, the supernatant containing the DNA was purified with Genomic-tip 100/G (Qiagen; #10243) with the Genomic DNA Buffer Set (Qiagen; #19060) following the manufacturer's instructions. To the resulting flow-through, 0.7 volumes of isopropanol were gently added, and the precipitated DNA was spooled with a glass hook through slow tube rotations, and resuspended in Elution Buffer (EB, Qiagen; #19086) overnight at 4°C.

For Col-0, we extracted HMW-DNA following a modified version of a published protocol (34) that included the addition of β-mercapto-ethanol during the lysis step and a phenol:chloroform purification step (35). Briefly, 300 mg of tissue powder was incubated for 45 min at 55°C in freshly prepared, pre-heated lysis buffer (1% sodium metabisulfite, 1% PVP40, 0.5 M NaCl, 100 mM Tris–HCl pH 8, 50 mM EDTA pH 8, 1.5% SDS, 2% β-mercapto-ethanol). The following steps were performed at room temperature. 60 μl of 20 mg/ml PureLink^TM^ RNAseA (Thermo Fisher Scientific; #12091021) was added to the lysate and incubated for 10 min. To precipitate proteins, 600 μl of 5 M potassium acetate was added to the samples followed by 2.4 ml of 25:24:1 (v/v/v) phenol:chloroform:isoamyl alcohol (ROTI; #A156.1) and incubated for 10 min with slow rotation. After centrifuging at 4400 g for 10 min, the upper phase was transferred to a new tube and mixed with 24:1 (v/v) chloroform:isoamyl alcohol for 10 min with slow rotation. Following a second centrifugation at 4400 g for 10 min, the upper phase was transferred to a new tube and two bead cleanups were performed to remove contaminants. The first cleanup was performed for 30–60 min under slow rotation with 1x volume of 0.4% solution of SeraMag SpeedBeads® Carboxyl Magnetic Beads (GE Healthcare; #65152105050450). After placing the tube on a magnet, the supernatant was discarded and beads were washed twice with 80% ethanol. Elution was performed with 50 μl EB (Qiagen) after incubation at 37°C for 15 min. The second cleanup was performed with 0.45× volume of AMPure PB magnetic beads (Pacific Biosciences; #100-265-900). After binding for 30 min under slow rotation, beads were placed on a magnet and washed twice with 80% ethanol. For elution, 45 μl EB (Qiagen) was added and incubated for 10–15 min under slow rotation.

### Long-reads library preparation

For the CLR library of Ey15-2, 10 μg of HMW-DNA that had been sheared twice with a needle (FINE-JECT 0.45 × 25 mm, LOT 14-13651; Henke Sass Wolf; #4710004525) was used to prepare double libraries with the SMRTbell Express Template Preparation Kit 2.0 (PacBio; #101-693-800 Version 01). The libraries were size-selected with the BluePippin system (SageScience) with a 30 kb cutoff in a 0.75% DF Marker U1 high-pass 30–40kb vs3 gel cassette (Biozym; #BLF7510). The library was sequenced with sequencing primer v4 and no pre-extension time on a single SMRT Cell (30 hours movie time) with the Sequel II system (PacBio) using the Binding Kit 2.0 (PacBio; #101-842-900).

For the HiFi library of Ey15-2, HMW-DNA (25 ng/μl) was separately sheared with 30 and 35 kb settings using a Megaruptor 2 instrument (Diagenode SA). Because the resulting average insert sizes were shorter than expected, approximately 19 and 24 kb, respectively, 10 μg of both sheared fractions were combined in equal amounts and used to prepare double libraries (PacBio; #101-853-100 Version 03) with the HiFi SMRTbell Express Template Prep Kit 2.0. The libraries were size-selected with the BluePippin system (SageScience) with 17 kb cutoff in a 0.75% DF Marker S1 High-Pass 6–10kb vs3 gel cassette (Biozym). The library was sequenced with sequencing primer v2 (PacBio, #101-847-900) and 4h of pre-extension time on a single SMRT Cell with the Sequel II system using the Binding Kit 2.0.

For the HiFi library of Col-0, HMW-DNA (120 ng/μl) was sheared twice (back and forth) with a gTUBE (Covaris; #520079) in an Eppendorf Centrifuge 5424 at 4,800 rpm (soft) for 3 × 1 min. Five μg of sheared DNA were used to prepare libraries using the HiFi SMRTbell Express Template Prep Kit 2.0 (PacBio; #100-938-900) with SMRTbell Barcoded Adapter bc1022 (‘CACTCACGTGTGATAT’) and SMRTbell Enzyme Clean Up Kit 2.0 (PacBio; #101-932-600). Since this library was multiplexed with another unpublished sample, we used the protocol ‘Procedure & Checklist’ (PacBio; #101-853-100 Version 04) with minor modifications. The two libraries were combined in equal amounts and size-selected with the BluePippin system (SageScience) with 10 kb cutoff in a 0.75% DF Marker S1 High-Pass 6–10kb vs3 gel cassette (Biozym; #BLF7510). The library pool was sequenced with sequencing primer v5 (PacBio; #102-067-400) and 2 h of pre-extension time on a single SMRT Cell with the Sequel II system using the Binding Kit 2.2 (PacBio; #101-894-200).

### DNA extraction and short-reads library preparation

DNA for PCR-free data was extracted with the DNeasy Plant Mini Kit (Qiagen; #69104) following the manufacturer's instructions from the same tissue sample (after grinding) as the one used for HMW-DNA extraction. 700 ng of DNA were fragmented using an S2 Focused Ultrasonicator (Covaris) with the following settings: intensity 5, 10% duty cycle, 200 cycles and 45 s treatment time. A library was prepared with the NxSeq AmpFREE Low DNA Library Kit (Lucigen; #14000-1) according to the manufacturer's instructions with one slight modification. Following adapter ligation and prior to the final bead-cleanup at the purification step, we introduced an additional bead-cleanup (0.6:1, bead:library ratio) to remove long inserts. Library concentration was measured with the Qubit 2.0 Fluorometer (Invitrogen). The insert size was estimated to be around 460 bp (including adaptors) with a High Sensitivity DNA Chip (Agilent; #5067-4626) on a Bioanalyzer 2100 instrument (Agilent). The library was sequenced with paired-end 150 bp reads to a coverage depth of about 166x on a HiSeq 3000 instrument (Illumina).

### Generation of optical map

*A. thaliana* plants of accession Ey15-2 were germinated *in vitro* and transferred to soil in flats. To minimize starch accumulation, plants were placed in the dark for 24 hours before tissue collection. Ultra-HMW DNA was isolated from young plants using a modified version of a published protocol (36), which is based on the Bionano DNA Plant Isolation kit (Bionano Genomics; #80003). Approximately 2 g of young, healthy, light-starved leaves were transferred to a 50 ml conical tube and incubated for 20 min in 60 ml ice-cold Bionano Fixing solution after adding 3.2 ml formaldehyde, followed by three 10 min washes in 60 ml ice-cold Bionano Fixing solution without formaldehyde. The resulting fixed tissue was placed in a chilled square Petri dish with 4.5 ml ice-cold Bionano Homogenization buffer supplemented with 1 μM spermine tetrahydrochloride, 1 μM spermidine trihydrochloride and 0.2% β-mercapto-ethanol. Leaves were manually chopped with a razor blade and transferred to a 50 ml conical tube, blended three or four times for 20 s in ice using a TissueRuptor (Qiagen) and filtered through 100 and 40 μM cell strainers. Nuclei and cell debris were pelleted by centrifugation at 3100 g, the supernatant decanted and the resulting pellet resuspended by swirling. Excess starch and cell debris in the original pellet were removed by low-speed centrifugation. The tube with the resuspended pellet was filled with fresh homogenization buffer, mixed by inversion and centrifuged for 2 min at 100 g with slow deceleration. The top three quarters of the supernatant were recovered by carefully decanting 35 ml into a new 50 ml tube, leaving excess contaminants at the bottom in the last 10–15 ml. This process was repeated two or three times until the supernatant was clear and the pellet was reduced in size. The nuclei in the supernatant were recovered by centrifugation at 3100 g and were resuspended in 55 μl cold Bionano Density Gradient Buffer. The tube containing the final resuspension was incubated at 43°C, mixed with 1× melted low-melting-point agarose equilibrated at 43°C and allowed to solidify after transferring to a plug mold. The agarose-embedded nuclei were incubated twice at 50°C in Bionano Lysis Buffer with added 8% (v/v) proteinase K (Puregene), for a total of 12–16 h. RNase A (Puregene) was added to a total of 2% (v/v) and the plugs were incubated for 1 h at 37°C. Plugs were washed four times for 15 min each in Bionano Wash solution, followed by five 15 min washes in TE Buffer. Finally, ultra-HMW DNA was eluted from the agarose by melting the plugs at 70°C for 2 min in a thermomixer, allowing the temperature to decrease gradually to 43°C, adding 2 μl agarase and incubating at 43°C for 45 min. The highly viscous DNA samples were further cleaned up by drop dialysis against TE buffer and quantified using a Qubit Fluorometer (Invitrogen).

Optical mapping was performed using the Direct labeling and stain approach (Bionano Genomics; DLS) as described (37), but using only 350–500 ng of ultra-HMW DNA per reaction. The labeled sample was loaded into a Saphyr G2.3 chip (Bionano Genomics), and molecules were separated, imaged, and digitized using a Saphyr Analyzer and Compute server (Bionano Genomics).

### Genome size estimation

To estimate the genome size of Ey15-2 from PCR-free reads, we employed two different methods starting from a dataset with pre-processed reads for which we trimmed remaining adapters from raw reads, removed low quality bases and discarded reads shorter than 75 bp (-q 20,15 –trim-n –minimum-length 75) with cutadapt v2.4 (38). For the *k*-mer based approach, we first aligned pre-processed reads to the chloroplast and mitochondrial genomes of TAIR10 and the bacteriophage phiX174 genome with bwa-mem v0.7.17 (39). We discarded reads that did not align to the nuclear genome with a series of Samtools v1.9 (40) commands. To obtain paired-reads alignments in which read1 was unmapped and read2 was mapped, we used ‘samtools view -b -f 4 -F 264’. Conversely, to obtain paired-read alignments in which read1 was mapped and read2 was unmapped, we used ‘samtools view -b -f 8 -F 260’. To retrieve pairs in which both reads were unmapped, we used ‘samtools view -b -f 12 -F 256’. We combined the outputs of the three previous steps with ‘samtools merge’, discarded supplementary alignments with ‘samtools view -b -F 2048’ and converted the BAM file to FASTQ format with bedtools ‘bedtools bamtofastq’ v2.27.1 (41). To count *k*-mers we employed the ‘count’ (-C -m 21 -s 5G) and ‘histo’ commands from Jellyfish v2.3.0 (42) with a *k*-mer size of 21. Finally, with an R-script from the findGSE tool (43) we estimated the genome size to be 143.12 Mb. For the mapping-based approach, we aligned with bwa-mem v0.7.17 (39) pre-processed reads to the ‘HiFi + CLR’ hybrid assembly (see below), to which we added the chloroplast and mitochondrial genomes of the TAIR10 reference genome and the bacteriophage phiX174 genome. We ran the Mapping-based Genome Size Estimation (MGSE) (44) tool, choosing as normalizing loci the Benchmarking Universal Single-Copy Orthologs (BUSCO) (45) ‘embryophyta_odb10’ gene set (*n* = 1375), and excluding the mitochondria, chloroplast and phiX174 genome from the calculations. The estimated genome size with this method was 145.28 Mb.

### CLR assembly

The CLR subreads BAM file was converted to FASTA format with SAMtools v1.7 (40) and subreads shorter than 10 kb (seq -L 10000) were discarded with seqtk v1.3 (https://github.com/lh3/seqtk). This file was used as input for Canu v2.0 (30) for assembly with a maximum input coverage of 200x and an estimated genome size of 140 Mb (canu -pacbio-raw <input-reads> genomeSize = 140mb maxInputCoverage = 200 correctedErrorRate = 0.035 utgOvlErrorRate = 0.065 trimReadsCoverage = 2 trimReadsOverlap = 500). To polish the assembled contigs, we aligned a 20% subset of the subreads larger than 10 kb with pbmm2 v1.0.0 (align –preset SUBREAD), and used GCpp v1.9.0 with the Arrow algorithm (PacBio tools; https://github.com/PacificBiosciences/pbbioconda).

### HiFi reads subsets

q20 High Fidelity (HiFi) reads were generated with the Circular Consensus Sequencing tool from PacBio, ccs v6.0.0 (–min-passes 3 –min-length 10 –max-length 60000 –min-rq 0.99). To study the impact of coverage in different HiFi assemblers, the original ∼107x q20 HiFi dataset was subsetted to 125×, 100×, 75×, 50×, 25× and 15× with rasusa v0.3.0 (46) (–genome-size 140 mb), equivalent to 101×, 81×, 60×, 40×, 20× and 12× effective coverage based on alignment to the TAIR10 reference genome. For each coverage subset, five replicates were generated using seed values 3, 19, 23, 54 and 70, resulting in 30 subsets.

To assess the impact of read length in different HiFi assemblers, we trimmed all reads in the original HiFi dataset, which had a median read length of 21.5 kb, with the command ‘trimfq’ from seqtk v1.3 (https://github.com/lh3/seqtk). By trimming 0, 1, 2, 3 and 4 kb from each end of the reads, we generated subsets with median read lengths of 21.5, 19.5, 17.5, 15.5 and 13.5 kb, respectively. Afterwards, reads shorter than 2 kb in the resulting subsets were discarded. The effective coverage based on alignment to the TAIR10 reference genome in the smallest read subset was slightly above 67×. Therefore, all sets were subjected to five replicates of downsampling to 85× with rasusa (46) as described above, resulting in a total of 25 subsets.

### HiFi assemblies

The original HiFi set along with 30 subsets of different coverages and 25 subsets of different read lengths were each assembled with HiCanu (31), FALCON (12,24), Hifiasm (32), Peregrine (33) and IPA (https://github.com/PacificBiosciences/pbipa). Identical commands were used for all different subsets per assembler.

HiCanu was used through Canu v2.0 (30,31) with a maximum coverage threshold above the read depth of all subsets (-assemble -pacbio-hifi genomeSize = 140m maxInputCoverage = 200). HiFi FALCON assemblies were run by executing the toolkit (12,24) distributed with the ‘PacBio Assembly Tool Suite’ v0.0.8 (falcon-kit 1.8.1; pypeflow 2.3.0; https://github.com/PacificBiosciences/pb-assembly). An example configuration file with detailed assembly parameters used in this study is provided in the dedicated GitHub for this study. The same input HiFi reads used for assembly were further mapped to the resulting contigs with pbmm2 v1.0.0 (align –preset CCS –sort), and polished with Racon v1.4.10 (47). The assemblies performed with Hifiasm (32) only needed the specification of a parameter for small genomes (-f0) and the disabling of purging of duplicated contigs recommended for inbred genomes (-l0). All Ey15-2 subsets were assembled with Hifiasm v0.13-r308, while the Col-0 sample was assembled with Hifiasm v0.16.1-r375 (32). Peregrine v1.6.3 (33) was run using the following command for all assemblies: ‘pg_run.py asm index_nchunk = 48 index_nproc = 48 ovlp_nchunk = 48 ovlp_nproc = 48 mapping_nchunk = 48 mapping_nproc = 48 cns_nchunk = 48 cns_nproc = 48 sort_nproc = 48 –with-consensus –shimmer-r 3 –best_n_ovlp 8’. PacBio's IPA v1.3.1 (https://github.com/PacificBiosciences/pbipa) was used in cluster mode (dist) and skipping phasin (–no-phase) for inbred genomes.

### Scaffolding with optical maps

Data visualization, map assembly, and hybrid scaffold construction were performed per manufacturer's recommendations using Bionano Access v1.5 and Bionano Solve v3.6 (https://bionanogenomics.com/support/software-downloads). The assembly was performed in pre-assembly mode using parameters ‘non-haplotype’ and ‘no-CMPR-cut’, without extend-split.

The resulting agp files of the hybrid scaffolds were manually curated to specifically discard: (i) complete super-scaffolds—and their associated contigs—of organellar DNA, (ii) complete super-scaffolds—and their associated contigs—of 45S rDNAs and (iii) isolated contigs ‘hybridizing’ to the 45S rDNA portion of otherwise larger super-scaffolds. A complete list of all super-scaffolds and contigs removed from the Bionano-based scaffolds is provided in Supplementary File 1. Similarly, these contigs were also added to the list of non-scaffolded contigs that was used for the analysis of contig breaks (see below). Edited agp files were converted to fasta format with the script ‘ragtag_agp2fasta.py’ from RagTag v1.1.1 (48). Super-scaffolds were assigned to their corresponding *A. thaliana* chromosome with the function ‘scaffold’ from RagTag v1.1.1 (48).

### Reference-based scaffolding

For the evaluation of accuracy and completeness, we scaffolded contigs >150 kb with RagTag v1.1.1 (48) (scaffold -q 60 -f 10000 -I 0.5 –remove-small) using a hard-masked version of TAIR10 as reference genome. For Col-0, the procedure differed slightly, and we scaffolded contigs >100 kb with RagTag v2.0.1 (48) (scaffold -q 60 -f 30000 -I 0.5 –remove-small), also using the hard-masked version of TAIR10 as reference. Since we observed that *in silico* scaffolding can be subject to biases due to structural variants distinguishing reference and target, we took the precaution of masking regions in the TAIR10 reference genome that could lead to misplacement of contigs. To this end, we used the function ‘bedtools maskfasta’ v2.27.1 (41) with ranges corresponding to our own annotation of centromeres, telomeres, organellar nuclear insertions and both 5S and 45S rDNAs (see section below). Since our annotation of centromeres was specific to the satellite repeat CEN180, we also masked large portions of the pericentromeric region in TAIR10 (Chr1:14309681–15438174, Chr2:3602469–3728277, Chr3:13586904–13870733, Chr3:14132986–14225247, Chr4:2919189–2981850, Chr4:3024926–3061554, Chr4:3194356–3263238, Chr4:3950509–4061755, Chr5:11184520–11316773, Chr5:11651274–12065554, Chr5:12807214–12870360).

### Assembly metrics

Contiguity, correctness (base-level accuracy) and completeness of the single CLR and all 280 HiFi assemblies were analyzed using identical commands. Because the total contig lengths of the different assemblies varied massively (particularly between assemblers), we used NG50 instead of N50 to evaluate contiguity. We defined NG50 as the sequence length of the shortest contig for which longer and equal length contigs cover at least 50% of the size of the TAIR10 reference genome (119.14 Mb; (2)). Scaffolded length, correctness and completeness metrics were estimated on scaffolded contigs, whether from *de novo* scaffolding with Bionano optical maps or reference-based scaffolding with RagTag. Therefore, depending on the scaffolding method, the exact values for the complete set (107x) differed slightly between Table 1 and Figure 2D and E. To estimate correctness and completeness, we used Merqury v1.1 (49), which compares *k*-mers in the *de novo* assemblies to those found in the raw PCR-free Illumina short reads. First, two *k*-mer databases with ‘*k* = 18’ were generated from Illumina paired-end reads with Meryl v1.3 (50) and combined with ‘meryl union-sum’. Merqury was run for each assembly using these *k*-mer counts as databases. Finally, genome-wide consensus quality (QV) and completeness scores were collected (Supplementary File 2). For the CLR and the five HiFi assemblies with the complete read set (Table 1), we also calculated BUSCO scores (v3.0.2; ‘-l embryophyta_odb10 -m genome -sp arabidopsis’) as an estimate of gene completeness (45). Additionally, LTR Assembly Index (LAI) scores (51) were estimated based on the total LTR sequence content, the mean LTR identity of the most complete assembly (HiFi-Hifiasm), and the *k*-mer based genome size estimate (LAI -totLTR 6.88 -iden 93.90 -genome_size 142000000) with LTR_retriever v2.9.0 (commit 460bb30) (52).

**Table 1.** Metrics of the CLR and five HiFi genome assemblies of *A. thaliana* Ey15-2

						Merqury		
Assembler	Total length [Mb]	Scaffolded length* [Mb]	Largest contig [Mb]	Contig NG50 [Mb]	BUSCO completeness*	Completeness*	QV*	LAI*
CLR Canu + Arrow	129.64	121.21	16.37	14.82	C:98.7% [S:98.0%,D:0.7%], F:0.3%,M:1.0%	98.72%	54.45	16.83
HiFi IPA	125.96	123.43	15.33	12.41	C:98.6% [S:97.9%,D:0.7%], F:0.3%,M:1.1%	98.43%	54.11	16.63
HiFi Peregrine	295.26	123.16	16.34	11.28	C:98.4% [S:97.7%,D:0.7%], F:0.4%,M:1.2%	98.59%	51.45	16.99
HiFi FALCON + Racon	140.60	136.09	34.35	12.44	C:98.5% [S:97.8%,D:0.7%], F:0.4%,M:1.1%	98.92%	52.83	19.35
HiFi HiCanu	234.77	135.57	34.36	16.33	C:98.4% [S:97.6%,D:0.8%], F:0.4%,M:1.2%	98.93%	57.56	18.49
HiFi Hifiasm	184.89	136.16	34.36	16.32	C:98.5% [S:97.7%,D:0.8%], F:0.4%,M:1.1%	98.94%	60.26	19.60

We define NG50 as the sequence length of the shortest contig for which longer and equal length contigs cover at least 50% of the size of the TAIR10 reference genome (119.14 Mb; (2)). Benchmarking Universal Single-Copy Orthologs (BUSCO) (45) scores were obtained with the ‘embryophyta_odb10’ set (*n* = 1375). Complete (C), single copy (S), duplicated (D), fragmented (F) and missing (M) genes are indicated. LTR Assembly Index (LAI) (51) values were estimated by specifying the same total LTR sequence content, mean LTR identity and genome size for all assemblies (see Materials and Methods). *Scaffolded length, BUSCO scores, Merqury's QV and completeness (49), and LAI values were computed on contigs scaffolded with Bionano optical maps.

### Gap inspection

To create the ‘HiFi + CLR’ hybrid assembly of Ey15-2, we used the ‘patch’ function (-f 10000 –remove-small –join-only) of RagTag v2.0 (48) with the HiFi-HiFiasm contigs as a target and the CLR-Canu contigs as a query. We used pbmm2 v1.3.0 to align the CLR (align –preset SUBREAD –best-n 1 –min-length 500) and HiFi reads (align –preset CCS –best-n 1 –min-length 500) to the new assembly, and IGV v2.6.3 (53) to visualize ‘patched’ loci.

To analyze gaps in our HiFi-Hifiasm assembly of Col-0, we aligned the contigs to a recently published assembly of the same accession (19) with minimap2 v2.17 (54) (-ax asm5) and inspected the loci where adjacent contigs break with IGV v2.6.3 (53). We summarized the results of these analyses in Supplementary File 1.

### Annotation and analysis of repetitive elements

We annotated repetitive elements in the CLR-Canu assembly, as well as in HiFi-Hifiasm, HiFi-HiCanu, HiFi-FALCON, HiFi-Peregrine and HiFi-IPA assemblies of Ey15-2 that were based on the complete HiFi set. First, we ran RepeatMasker v4.0.9 (http://www.repeatmasker.org) (-cutoff 200 -nolow -gff -xsmall) using a custom library that included six CEN180 repeat clusters (55), three consensus 5S rDNA units (56), a reference 45S rDNA unit (57), and the telomere motif ‘[CCCTAAA]_60_’. With minimap2 v2.16 (54) (-cx asm5) and the organellar genomes from the TAIR10 reference (2), we identified organellar sequences in our assemblies. The gff2 and paf outputs of RepeatMasker and minimap2, respectively, were reformatted to gff3. Separately, transposable elements (TEs) and other repeat regions were annotated with Extensive de-novo TE Annotator (EDTA) v1.9.7 (58) (–step all –sensitive 1 –anno 1 –overwrite 1), which combines various TE annotation tools such as LTRharvest, LTR_FINDER, LTR_retriever, TIR-Learner, HelitronScanner, TEsorter (52,59–65). Finally, to combine all previous annotations, a series of ‘merge’ and ‘intersect’ commands from bedtools v2.27.1 (41) were used to avoid any overlap between—sometimes—conflictive repetitive elements with the following hierarchy: organellar sequence > rDNAs > TEs.

To contextualize the contribution of these repetitive elements to the assemblies, we counted their cumulative length separately for scaffolded and non-scaffolded contigs as determined from the scaffolding with optical maps. For the analysis of contig breaks, only contigs >10 kb and only 2 kb from the contig ends were considered.

For the analysis of centromere and 5S rDNA copy number variation, we chose the ‘HiFi + CLR’ hybrid assembly for Ey15-2 and the HiFi-Hifiasm assembly of Col-0. In addition, we downloaded the two most recent Col-0 assemblies of Naish *et al.* (19) from https://github.com/schatzlab/Col-CEN/tree/main/v1.2 and of Wang *et al.* (66) from https://ngdc.cncb.ac.cn/gwh/Assembly/21820/show. To estimate the number of 5S rRNA copies before assembly of HiFi samples, we ran RepeatMasker v4.0.9 (http://www.repeatmasker.org) (-cutoff 200 -nolow -gff -xsmall) directly on q20 HiFi reads using a custom library that included the canonical sequence of rRNA gene subunits, and counted the number of 5S rRNA gene matches >100 bp (205,573 and 363,615 for Ey15-2 and Col-0, respectively). We normalized these numbers by the genome-wide read depths obtained with samtools (40) (coverage -r Chr3:1–10000000) after aligning the HiFi reads to their own references with minimap2 v2.17 (54) (-ax asm20), which was 110.352 for Ey15-2 and 121.864 for Col-0.

### Analysis of collapsed and expandable sequences

Long-reads (HiFi or CLR) were aligned with pbmm2 v1.3.0 (align –sort –log-level DEBUG –preset SUBREAD –min-length 5000) to their corresponding chromosome scaffolds. Unmapped reads, as well as secondary and supplementary alignments were removed with samtools v1.9 (view -b -F 2308 < input.bam > Chr1 Chr2 Chr3 Chr4 Chr5) (40). The resulting bam file was used to determine across the entire genome the coverage of primary and secondary alleles with NucFreq v0.1 (–minobed 2) (67). The distribution of coverage with HiFi reads along the chromosomes (Supplementary Figure S4) shows a uniform coverage increase over all centromere regions in the HiFi-FALCON, HiFi-HiCanu and HiFi-Hifiasm assemblies of Ey15-2. This coverage increase was restricted to the primary allele and, importantly, not accompanied by an increase of the secondary allele, as would have been the case for assembly collapses of divergent duplicated regions. Instead, the pattern is reminiscent of what has been observed for specific satellite classes in the human telomere-to-telomere genome, potentially due to biases introduced during sample preparation or sequencing, as previously suggested (68). In support, coverage with CLR subreads did not increase at the Ey15-2 centromeres (Supplementary Figure S5), which was also the case for the HiFi reads of Col-0 aligned to its own Hifiasm assembly (Supplementary Figure S9). We therefore identified collapsed and expandable sequences in Ey15-2 assemblies with the tool Segmental Duplication Assembler (SDA v0.1.0) (67) using the bam file of aligned CLR subreads. Coordinates overlapping centromeres, 45S rDNA, 5S rDNAs, and organellar nuclear insertions were identified with the ‘intersect’ command from bedtools v2.27.1 (41).

### Data manipulation and plotting

Most analyses and data visualization was done with R v4.0.2 (https://www.r-project.org) and RStudio v1.3.1073 (https://www.rstudio.com). R packages ‘ggplot2’ (https://ggplot2.tidyverse.org), ‘ggh4x’ (https://github.com/teunbrand/ggh4x), ‘plyr’ (69), ‘data.table’ (https://github.com/Rdatatable/data.table) were instrumental for this study. Alignments between assemblies were visualized with AliTV (70) using the MiniTV wrapper (https://github.com/weigelworld/minitv). Pericentromeric regions were visualized with StainedGlass v0.4 (window = 5000 mm_f = 10000) (71).

## RESULTS

**Figure 1.**
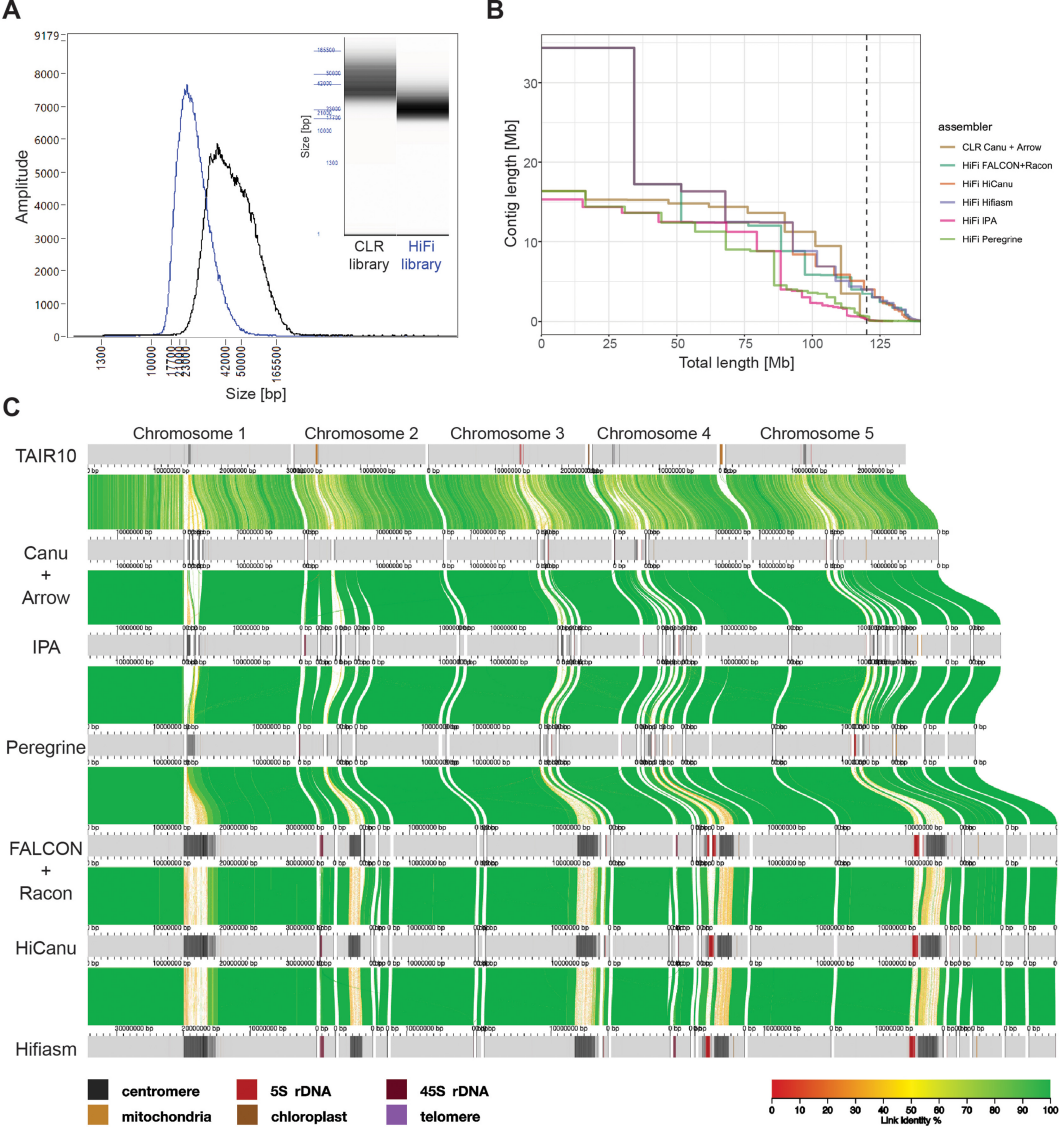
Comparison of different PacBio libraries and assemblers. (**A**) Insert size distribution of the CLR (black) and HiFi (blue) libraries after size-selection on the BluePippin instrument as measured on a Femto Pulse System. (**B**) Contiguity plot comparing the CLR and five HiFi assemblies using the complete dataset. For each assembly, the cumulative contig length (ordered from largest to shortest) is plotted over the estimated genome size of *A. thaliana* accession Ey15-2 (∼143 Mb). The vertical dashed line indicates the size of the TAIR10 reference genome (119.14 Mb). (**C**) Alignment of the TAIR10 reference genome and the contigs of the CLR and five HiFi assemblies visualized by AliTV (70). Co-linear horizontal gray bars represent chromosomes or contigs, with sequence annotated as repetitive elements (centromeres, 5S and 45S rDNAs, telomeres, mitochondrial and chloroplast nuclear insertions) indicated by the colors shown on the bottom left. Only Bionano-scaffolded contigs >150 kb are shown. Distance between ticks equals 1 Mb. Colored ribbons connect corresponding regions in the alignment.

To compare the performance of PacBio's two long-read sequencing platforms, we generated CLR (subread coverage ∼1006×) and HiFi libraries (q20 HiFi read coverage ∼107×) starting from the same high molecular weight DNA extraction of a pool of individuals of the *A. thaliana* natural accession Ey15-2 (accession ID 9994; CS76399) (Figure 1A). We produced an optical map with the Bionano Direct Label and Stain (DLS) technology (molecule coverage ∼781×) to validate and scaffold the main assemblies. To evaluate completeness and accuracy of all assemblies and to estimate the genome size of Ey15-2, we made use of Illumina PCR-free paired-end reads (coverage ∼166×).

### Performance of the assembler of choice

To assemble contigs with the CLR dataset, we used Canu with a maximum input coverage of 200×, only using subreads larger than 10 kb, and polished the resulting assembly with Arrow (72), also using 200x of the initial long-reads. The resulting contigs had an NG50 of 14.82 Mb, on par with the best published *A. thaliana* CLR contigs (13–18).

With the HiFi dataset, we compared the performance of five assemblers: FALCON (24), HiCanu (31), Hifiasm (32), Peregrine (33), and Pacbio's Improved Phased Assembler (IPA; github.com/PacificBiosciences/pbipa). With the complete q20 HiFi dataset (∼107×), which has a median read length of 21.5 kb, we observed substantial differences in contig continuity for the different assemblers (Table 1). Only HiFi-Hifiasm and HiFi-HiCanu, both with 16.33 Mb, showed a higher NG50 than the CLR contigs. However, NG50 alone may not reflect the most noticeable differences in continuity between assemblers. HiFi-IPA and HiFi-Peregrine largest contigs, 15.33 and 16.34 Mb, respectively, were comparable to the largest CLR-Canu contig (16.37 Mb), which represents an entire chromosome arm (Figure 1B). In contrast, HiFi-FALCON, HiFi-HiCanu and HiFi-Hifiasm all assembled a 34.36 Mb contig that corresponds to the telomere-to-telomere assembly of chromosome 1 in *A. thaliana* (Figure 1C). The second largest contig was also exclusively assembled by those three assemblers. With 17.2 Mb, it spans the upper arm of chromosome 3, presumably the entire centromere, and part of the other arm (Figure 1C). Similarly, the third largest contig of 16.33 Mb, only achieved by HiFi-Hifiasm and HiFi-HiCanu, corresponds to the upper arm of chromosome 5, presumably encompassing the complete centromere, and part of the lower arm (Figure 1C).

The total contig lengths of the different assemblers varied massively (Table 1), even among the HiFi methods, which had as input the exact same read set. Therefore, to evaluate accuracy and completeness on a more level playing field, we generated hybrid scaffolds of nuclear chromosomes for each of the described contig sets with Bionano optical maps. The scaffolded length of the different assemblers still differed by up to 14.95 Mb, equivalent to over 10% of the estimated genome size (see below), with the CLR-Canu, HiFi-IPA and HiFi-Peregrine assemblies at the low end, and the HiFi-HiCanu, HiFi-FALCON and HiFi-Hifiasm at the upper end (Table 1). By comparing *k*-mers in the *de novo* assemblies to those in the raw PCR-free Illumina short reads, the Merqury tool can estimate base-level accuracy and completeness (49). The HiFi-Hifiasm assembly had the highest accuracy, with a consensus quality (QV) score of 60.3, followed by HiCanu (QV 57.6). In contrast, the HiFi assemblers HiFi-IPA (QV 54.1), HiFi-Peregrine (QV 51.5) and HiFi-FALCON (QV 52.8) were all below the accuracy of the CLR-Canu assembly (QV 54.5). Meanwhile, *k*-mer based completeness was less informative, as there was limited variation among assemblies, despite the massive variation in scaffolded length (Table 1). This was due to Merqury counting distinct *k*-mers found in the reads, regardless of their copy number (49). Similarly, the assessment of gene content of the assemblies with the widely used Benchmarking Universal Single-Copy Orthologs (BUSCO) score (45), although high (>98.4%), showed little difference among assemblies (Table 1). In contrast, the LTR Assembly Index (LAI), a standardized metric based on the detection of intact long terminal repeat (LTR) retrotransposons (51), ranks HiFi-Hifiasm and HiFi-FALCON as the assemblers that are superior in terms of LTR content, followed closely by HiFi-HiCanu, with the remaining assemblers, including CLR-Canu, being considerably worse (Table 1). Therefore, practically all assemblers were successful in the non-repetitive fraction of the genome, but the repetitive regions deserved special consideration (see below).

### Impact of coverage

**Figure 2.**
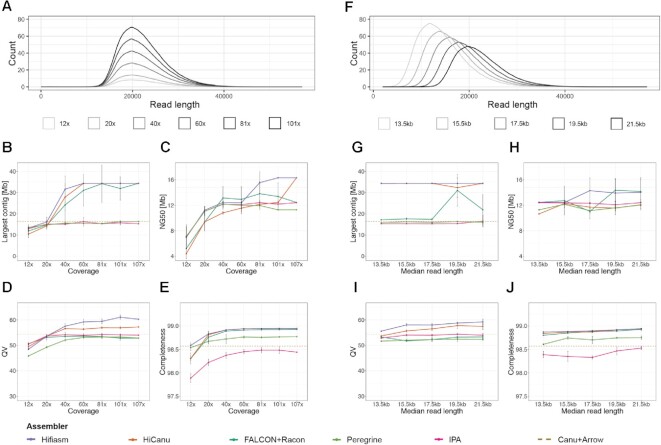
Impact of coverage and read length on assembly metrics. (**A**) Read length distribution of subsets of HiFi reads with varying coverages, 12×, 20×, 40×, 60×, 81× and 101×. (**B**) Largest contig as a function of input coverage. (**C**) Contig NG50 as a function of input coverage. We define NG50 as the sequence length of the shortest contig for which longer and equal size contigs cover at least 50% of the size of the TAIR10 reference genome (119.14 Mb) (2). (**D**) Consensus quality (QV) estimated by Merqury (49) as a function of input coverage. (**E**) *k*-mer completeness estimated by Merqury (49) as a function of input coverage. QV and completeness were computed after reference-based scaffolding with RagTag (48). (**F**) Read length distribution of subsets of HiFi reads with varying median read lengths, 13.5, 15.5, 17.5, 19.5 and 21.5 kb. (**G**) Largest contig as a function of median read length. (**H**) Contig NG50 as a function of median read length. (**I**) QV as a function of median read length. (**J**) *k*-mer completeness as a function of median read length.

Since HiFi technology supports barcodes to allow sequencing of several samples per SMRT Cell, it might often be more cost-effective to generate less read depth for *de novo* assemblies. To simulate data sets with decreasing coverage, starting from our complete q20 HiFi dataset at 107×, we generated five random subsets each for 101×, 81×, 60×, 40×, 20× and 12× coverage (Figure 2A). Each of the 25 subsets of reads was assembled with all five HiFi assemblers investigated in this study.

Both HiFi-Hifiasm and HiFi-HiCanu successfully assembled the same largest contig (∼34.4 Mb) in all replicates of subsets down to 60× coverage (Figure 2B). At 40× coverage, HiFi-Hifiasm failed to assemble this contig in one out of five replicate subsets, while with HiFi-HiCanu the contig broke in three of the replicates (Supplementary Figure S1). The lower continuity in HiFi-HiCanu when compared to HiFi-Hifiasm was also manifested in how often the second and third longest contigs were assembled, which is reflected by the progressive drop in NG50 at lower coverages (Figure 2C). Although HiFi-FALCON successfully assembled the three longest contigs in some replicates of subsets down to 40× coverage (Figure 2B), NG50 declined already at higher coverage than with HiFi-Hifiasm (Figure 2C). In addition, assemblies with HiFi-FALCON were more inconsistent across replicate subsets, to the degree that in two replicates of subset 81x chimeric contigs were formed (Supplementary Figure S2a, b). Nevertheless, HiFi-FALCON still performed better than both HiFi-Peregrine and HiFi-IPA with respect to both continuity metrics. When compared to the CLR-Canu assembly, however, only HiFi-HiCanu with the full set and HiFi-Hifiasm with coverages of at least 81× had a superior NG50 (Figure 2C).

After scaffolding, this time with RagTag (48), a reference-based scaffolding tool, we evaluated accuracy and completeness as described before. For all assemblers, QV scores were largely unaffected in subsets down to 40× coverage (Figure 2D), with HiFi-Hifiasm leading and HiFi-HiCanu coming in second. At 20× coverage, HiFi-Hifiasm and HiFi-HiCanu base-accuracy dropped to lower levels, albeit still comparable to all other assemblers, while HiFi-Peregrine QV scores fell below 50. At 12x coverage, QV scores further dropped for all HiFi assemblers, with scores for HiFi-Hifiasm and HiFi-HiCanu falling below 50. *k*-mer completeness was largely unaffected in subsets down to 20× coverage, but with 12× coverage all HiFi assemblers experienced drops for this metric, with HiFi-HiCanu, HiFi-FALCON and HiFi-IPA being the most affected assemblers at the lowest coverage (Figure 2E). Overall, HiFi-Hifiasm and HiFi-HiCanu stood out as the best assemblers across all metrics. In addition, HiFi-Hifiasm was more consistent in continuity and base quality, with compromises only apparent in some replicates of subsets with 60x and lower coverage.

### Impact of read length

The recommended insert size for HiFi libraries is 15–18 kb, but to test the limits of this technology, we generated a q20 HiFi dataset with a median read length of 21.5 kb and N50 of 22.58 kb. This enabled us to simulate datasets—also five replicates each—with decreasing median insert sizes in steps of 2 kb down to 13.5 kb, to evaluate the impact of read length on various assembly metrics (Figure 2F). Due to the dependence on coverage observed before, all subsets were reduced to the highest possible common coverage (∼67×).

Both HiFi-Hifiasm and HiFi-HiCanu successfully assembled the largest contig representing chromosome 1 in nearly all replicates of the different median read lengths, except for HiFi-HiCanu at 19.5 kb median read length (Figure 2G). HiFi-FALCON assembled the largest contig in half of the replicates of the two largest read length subsets, and failed to assemble it for all subsets with a median read length of 17.5 kb and below (Supplementary Figure S3). Similar to the situation observed in the coverage subsets, HiFi-FALCON produced a chimeric contig in one replicate of the subsets with median read length of 19.5 kb (Supplementary Figure S2c). The average NG50 values produced by all HiFi assemblers in all subsets were below the one achieved with CLRs (Figure 2H), which reflects the difficulty of assembling the second and third largest contigs (Supplementary Figure S3). HiFi-Hifiasm and HiFi-FALCON achieved higher average NG50 than the other HiFi assemblers for the two largest read length subsets, but NG50 dropped for HiFi-FALCON at 17.5 kb, and for HiFi-Hifiasm at 15.5 kb (Figure 2H). Both HiFi-Peregrine and HiFi-IPA did not show much variation either for the largest contig or NG50 across different read length subsets, and remain the HiFi assemblers performing the poorest for these metrics. Base-level accuracy and completeness for each assembler were very similar across all read length subsets, and the order mirrored what was observed for the complete read set (Figure 2I, J). In conclusion, read length (down to a median of 13.5 kb) does not appear to have as much impact as coverage in most assembly metrics for the *A. thaliana* genome.

### Repetitive elements in scaffolds and contigs

**Figure 3.**
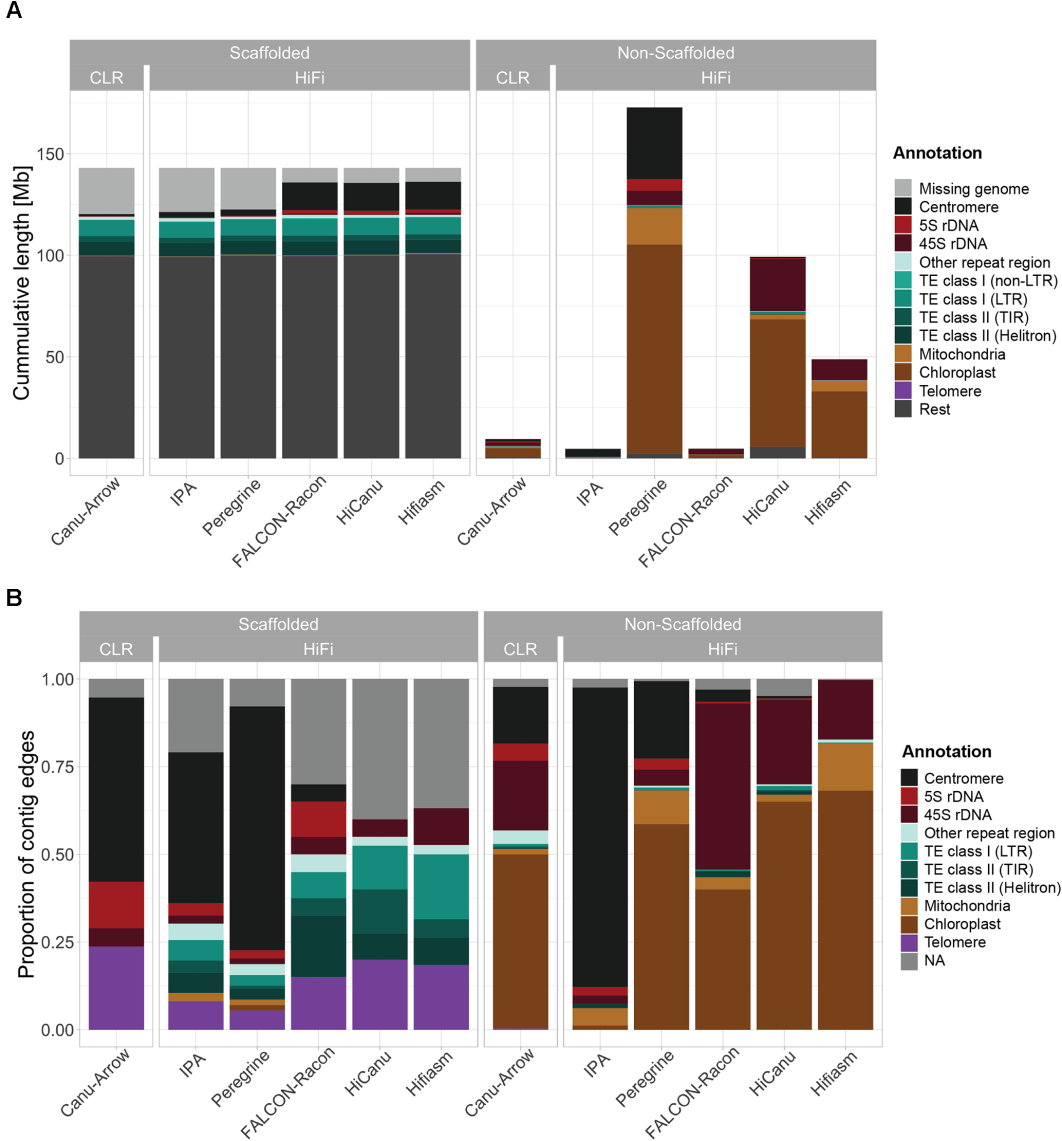
Repetitive elements in scaffolded and non-scaffolded contigs. (**A**) Stacked barplot of the cumulative length of various repetitive elements split into the scaffolded nuclear genome (left) and non-scaffolded contigs (right) for the CLR and HiFi assemblies. The height of the bars for the scaffolded genome is 143 Mb, the *k*-mer based genome size estimate by findGSE (43). (**B**) Fractions of the repetitive element found first within 2 kb of each contig edge in scaffolded contigs (left) and non-scaffolded contigs (right).

To characterize the contribution of different genetic elements to the scaffolded genome for each of the assemblers, we annotated the repetitive elements in all contigs generated from the complete q20 HiFi dataset: transposable elements (TEs), centromeres, telomeres, 5S and 45S ribosomal RNA genes (rDNAs), as well as chloroplast and mitochondrial genome DNA insertions. In addition, using Illumina PCR-free short reads, we estimated the nuclear genome size of the Ey15-2 accession to be 143 Mb according to a *k*-mer based method (43) or 145 Mb based on a mapping-to-reference approach (44). Notably, the amount of non-repetitive sequence (understood as everything that was not annotated as a repetitive element) were very similar in the contigs successfully scaffolded with optical maps for the CLR and the HiFi assemblies (Figure 3A). While for the CLR the total non-repetitive sequence was 99.43 Mb (69.47% of the *k*-mer based genome size estimate), for the HiFi assemblies it ranged from 98.99 Mb (69.16%) in HiFi-IPA to 100.47 Mb in HiFi-Hifiasm (70.20%). Even when adding telomeres, organellar insertions and TEs to the non-repetitive sequence, this length added up to only 118.97 Mb (83.12%) in the CLR-Canu assembly, while in the HiFi assemblies it ranged from 118.3 Mb in HiFi-IPA (82.65%) to 119.95 Mb (83.81%) in HiFi-Hifiasm (Figure 3A). These values were remarkably similar to the total length of 119.14 Mb of the TAIR10 reference genome (2).

The substantial differences in the total length of nuclear scaffolds between technologies or assemblers are explained only when considering 5S rDNAs and centromeres. For the CLR-Canu assembly, we were only able to scaffold 159 kb of 5S rDNAs and 1.08 Mb of centromeres. Similar to the situation with other assembly metrics, performance of both HiFi-Peregrine and HiFi-IPA was closer to CLR-Canu than to the other HiFi assemblers. On the other hand, HiFi-FALCON, HiFi-HiCanu and HiFi-Hifiasm nuclear scaffolds contained very similar amounts of 5S rDNA arrays, 1.64–1.68 Mb, and of centromeres, 13.63–13.69 Mb. To investigate the reliability of our assemblies in these repetitive regions, we analyzed potentially collapsed and expandable sequences in the scaffolded assemblies (67,73). According to annotations of repeat features in the assemblies, 5S rDNAs and centromeres did not appear to contribute substantially to the collapsed sequences in the HiFi-FALCON, HiFi-HiCanu and HiFi-Hifiasm nuclear scaffolds (Supplementary Figure S6). In contrast, centromeres comprised most of the true collapsed regions in the CLR-based assembly. Therefore, the access to Mb-scale centromeric sequence and 5S rDNA arrays is what most clearly differentiates the most complete HiFi scaffolded assemblies from the CLR-based one (Figure 3A).

Nevertheless, even the largest scaffolded assemblies, i.e. Hifiasm, HiCanu and FALCON, do not reach the *k*-mer based genome size estimate; for these, there remain 6.94–7.52 Mb to be explained. To account for the missing sequence, we examined the non-scaffolded contigs. Their cumulative length per assembly (range 4.67–172.74 Mb) varied much more dramatically than their scaffolded counterpart (Figure 3A). Most of these discrepancies can be attributed to variation in organellar contig lengths and numbers. Similarly, the various assemblers produced discordant amounts of non-scaffolded sequence annotated as 45S rDNAs, the length of which did not correspond to the differences between the genome size estimate and the lengths of scaffolded contigs for each assembly (Supplementary Figure S7). Notably, for the HiFi-Hifiasm assembly, with 10.36 Mb of non-scaffolded 45S rDNA, representing 96% of the non-scaffolded sequence after removing organellar DNA, this value differed only by 3.42 Mb. To generate an independent 45S rDNA copy number estimate, we used a mapping-to-reference approach with Illumina PCR-free short reads (74), and estimated 1055 18S rRNA gene copies per haploid genome. Assuming 10.7 kb per 45S rDNA unit, this would equate to 11.28 Mb. Coincidentally, the total amount of scaffolded and non-scaffolded 45S rDNA added up to 11.3 Mb. However, it is important to consider that since the non-scaffolded contigs consisting of 45S rDNA are not anchored to the assembled genome by non-repetitive sequence, we can currently not validate whether they present all unique sequence blocks and what their orientation is. Unfortunately, when it comes to 45S rDNA clusters in *A. thaliana*, the high quality optical maps generated with the Bionano DLS technology are of limited use. This is due to the recognition sequence of the non-nicking enzyme DLE-1 (CTTAAG) occurring three times within 949 bp in the highly conserved 25S rRNA gene (one of the three rRNA components of each 45S rDNA unit), while there are no occurrences in the more variable internal or external transcribed spacers of a reference 45S rDNA unit (75). This makes optical maps uninformative at these loci, in turn impeding the reliable construction of hybrid scaffolds.

### Where do contigs break?

To investigate in more detail the genetic elements that may cause contigs to break, we determined what type of repetitive element was closest to each contig edge, considering the first 2 kb from each edge. In an ideal case of complete telomere-to-telomere contigs and with five nuclear chromosomes, one would expect ten contig edges identified as telomeric repeats in *A. thaliana*. In the CLR-Canu assembly, centromeric sequences were identified in more than half of the scaffolded contig edges (Figure 3B). Similarly, in the HiFi-Peregrine and HiFi-IPA assemblies, centromeric sequences at scaffolded contigs edges were found more often than any of the other repetitive elements (Figure 3B). In contrast, in the HiFi-FALCON assembly, only two scaffolded contig edges contained centromeric sequences while neither the HiFi-HiCanu nor the HiFi-Hifiasm contig breaks seemed to be due to centromeric sequence.

The next problematic repetitive elements for scaffolded contig edges in the CLR-Canu assembly were 5S rDNAs, followed by 45S rDNAs. At scaffolded contig edges, 5S rDNAs were also present in HiFi-IPA, HiFi-Peregrine and HiFi-FALCON assemblies, but not in HiFi-HiCanu and HiFi-Hifiasm. Regardless of the sequencing technology or assembler, all contigs that correspond to the upper arms of chromosomes 2 and 4 broke at the subtelomeric 45S rDNA repeats (76). Different from the CLR assembly, all HiFi assemblies contain TEs in a substantial fraction of their scaffolded contigs edges (Figure 3B). We explain the underlying cause of these and most other contig breaks by analyzing more in detail the HiFi-Hifiasm assembly in the following section.

### In the quest of telomere-to-telomere assemblies

A major goal for *de novo* genome assembly projects is to achieve chromosome-level, telomere-to-telomere assemblies. Generally, orthogonal approaches such as Hi-C chromosome contact information or optical maps are regarded as necessary to build confidence in the assembly (29). We compared whether this goal is within reach for our CLR assembly and our best HiFi (Hifiasm) assembly, by combining them with optical maps.

**Figure 4.**
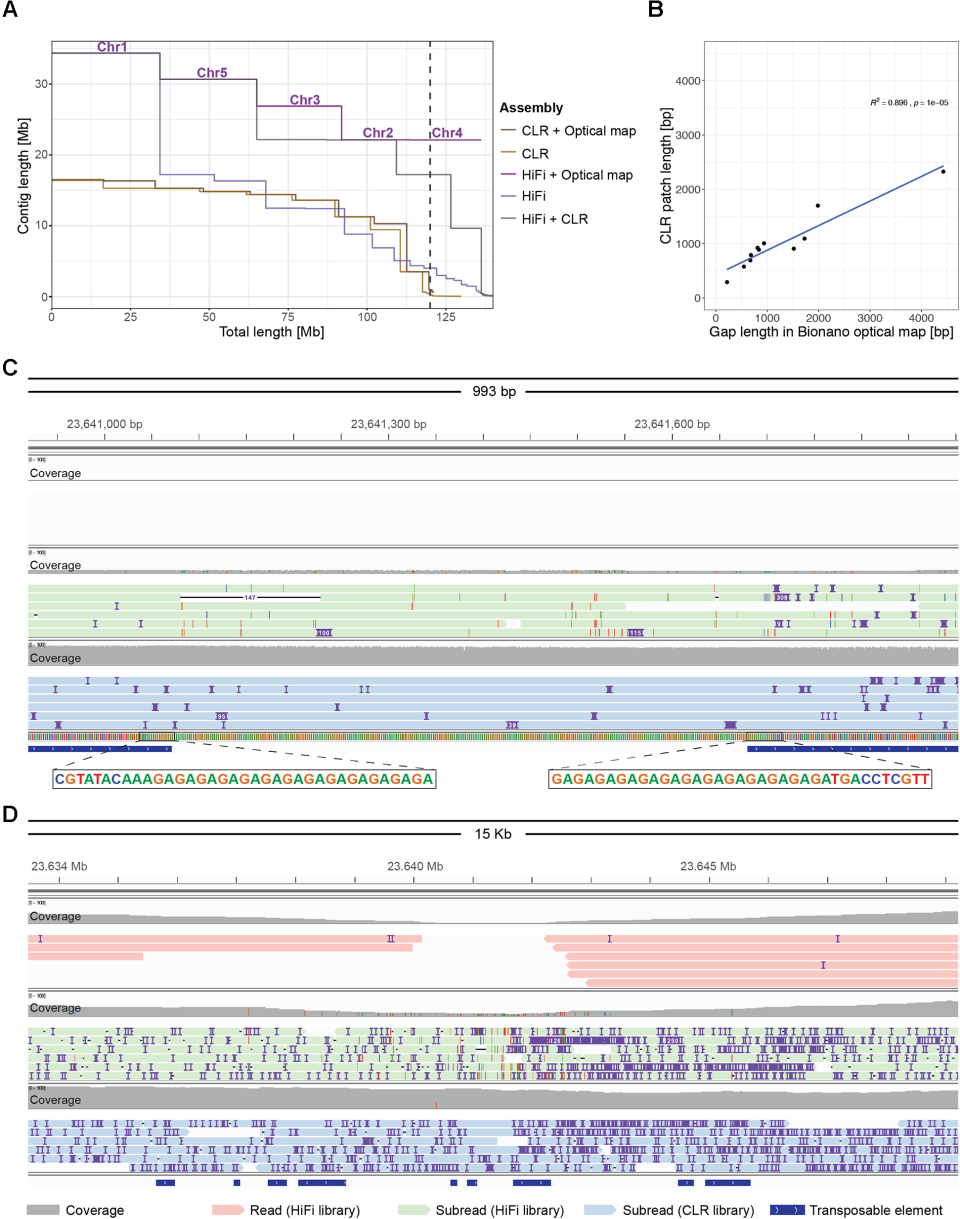
Hybrid assemblies and features of gaps. (**A**) Contiguity plot comparing the CLR-Canu and HiFi-Hifiasm assemblies alone, combined with RagTag ‘patch’ (48) or as hybrid scaffolds with Bionano optical maps. For each assembly, the cumulative contig—or scaffold—length (ordered from largest to shortest) is plotted over the estimated genome size of *A. thaliana* accession Ey15-2 (∼143 Mb). The vertical dashed line indicates the size of the TAIR10 reference genome (119.14 Mb). For the assembly that achieved ‘telomere’-to-telomere status (HiFi + optical map), chromosome numbers are indicated on top of the scaffold lines. (**B**) Correlation of gap lengths estimates between Bionano optical maps and CLR ‘patches’ introduced in the HiFi assembly by RagTag (48). (**C**) Visualization with IGV (53) of aligned HiFi reads (in red; top), subreads from the same HiFi library (in green; middle), and CLRs (in blue; bottom) over Chr5:23640917–23641913, a locus in the HiFi + CLR hybrid assembly ‘patched’ with sequences from the CLR-Canu assembly. (**D**) Zoom out of (C).

The CLR-Canu assembly scaffolded with optical maps and without the aid of reference information did not achieve a single chromosome-level assembly. Instead, the outcome from CLR-Canu combined with optical maps was a collection of ten hybrid scaffolds, each of which corresponded to a complete chromosome arm, with only three being slightly larger than the original contigs, plus two additional hybrid scaffolds with partial centromeres (Figure 4A). In fact, only very seldom do Bionano DLS optical maps span complete *A. thaliana* centromeres (1001G + Project). For species for which there is a reference genome available, such as *A. thaliana* TAIR10 (2), this limitation is not an issue, since reference-based scaffolding methods can be used to assign scaffolds to chromosomes. However, for species without a reference genome, Hi-C might be a better alternative for identifying chromosome arm-sized contigs that come from the same chromosome.

On the other hand, combining the HiFi-Hifiasm assembly with optical maps produced five ‘telomere’-to-telomere hybrid scaffolds (Figure 4A). The quotes in ‘telomere’ indicate that the upper arms of chromosomes 2 and 4 began with a few dozens units of subtelomeric 45S rRNA genes, rather than telomeric repeats, with the true telomeres presumably being on the other side of the not completely assembled 45S rDNA arrays. As shown in the analysis of contig breaks, all centromeres were complete in the HiFi-Hifiasm assembly (Figure 3B). The remaining six fragmented chromosome arms (Figure 1C) were properly scaffolded, although with fourteen gaps. From these, twelve gaps were estimated to be 217–6900 bp long. Two gaps were caused by contig overlaps not being properly resolved by Hifiasm in chromosomes 2 and 5. Contrary to the contig overlaps in chromosome 5 (Supplementary Figure S8a), the optical map indicated that one of the contig edges in chromosome 2 was inconsistent for DLE-1 recognition sites (Supplementary Figure S8b). The conflicting contig edge contained two 45S rDNA units supported by a single—likely chimeric—HiFi read. Upon removal of this read and further re-assembly, the resulting scaffold contained a normal gap at this position.

Given that the breaks in the CLR-Canu and the HiFi-Hifiasm contigs mostly did not overlap (Figure 1C), we combined both assemblies by ‘patching’ the most complete HiFi contig set with the CLR-Canu contigs using RagTag (48). This resulted in four ‘telomere’-to-telomere scaffolds, and only chromosome 3 split into two scaffolds (Figure 4A), which were separated by a gap estimated to be 6,900 bp according to the optical map (Supplementary Figure S8c). The pair of overlapping HiFi contigs corresponding to chromosome 5 was also identified and corrected by RagTag, which removed 7 bp (Supplementary Figure S8d). The CLR assembly only contributed a total of 12 049 bp across twelve ‘patches’, ranging from 290 to 2326 bp, largely in agreement with the gap sizes previously estimated with the optical map (Figure 4B; Supplementary File 1). A closer examination of these ‘patches’ revealed that all consisted of either GA/TC or GAA/TTC low-complexity repeats, and not TEs, as originally thought (Figure 3B). The presence of such repeats was supported by the CLR subreads, which spanned the complete region without a noticeable drop in coverage (Figure 4C). In contrast, q20 HiFi reads showed a drop in coverage extending for several kilobases around the low-complexity repeats (Figure 4D), which were generally not covered by any read—or by a single read in three out of the twelve instances. We also asked whether raw subreads from the HiFi library (before the circular consensus sequence step that generates HiFi reads) also experience coverage drops in these regions. This was indeed the case, although to a lesser extent than for Hifi reads (Figure 4C and D). The only major experimental difference in the preparation of CLR and HiFi libraries was the use of a different sequencing primer version and the introduction of four hours of polymerase pre-extension time during the sequencing of the Ey15-2 HiFi library (see Materials and Methods). The observation that GA/TC low-complexity regions are preferentially spanned in sequencing runs without pre-extension suggests that the responsible mechanism for reduced HiFi coverage in these regions relates to polymerase behavior in sequencing passes beyond the first one.

Coverage bias of HiFi chemistry at GA/TC low-complexity repeats has been previously noticed for four out of twelve gaps of a human X chromosome (31). To investigate whether this particular class of low-complexity repeats is responsible for contig breaks in a different *A. thaliana* genome, we sequenced with HiFi reads and assembled with Hifiasm a single individual of the accession Col-0 (accession ID 6909; CS76778; Supplementary Figure S9). The reference-based scaffolds contained only nine gaps. A comparison of our HiFi Col-0 assembly with the TAIR10 reference genome (2) and two recently published Col-0 assemblies (19,66) confirmed that eight of the nine gaps in our HiFi Col-0 (range: 601 to 1,861 bp) also occurred at GA/TC or GAA/TTC repeats (Supplementary File 1), with the remaining gap consisting of an unresolved 42 895 bp overlap between two contigs when compared to one of the recent assemblies (19). That contigs breaks in these *A. thaliana* long-read assemblies were mostly due to GA/TC low-complexity repeats (86% and 89% in the Ey15-2 and the Col-0 assemblies, respectively) points to a current limitation of HiFi reads. Given the relatively small sizes of the gaps, this is, however, only a minor weakness of this technology.

### Natural variation in centromeres and 5S rDNA clusters

**Figure 5.**
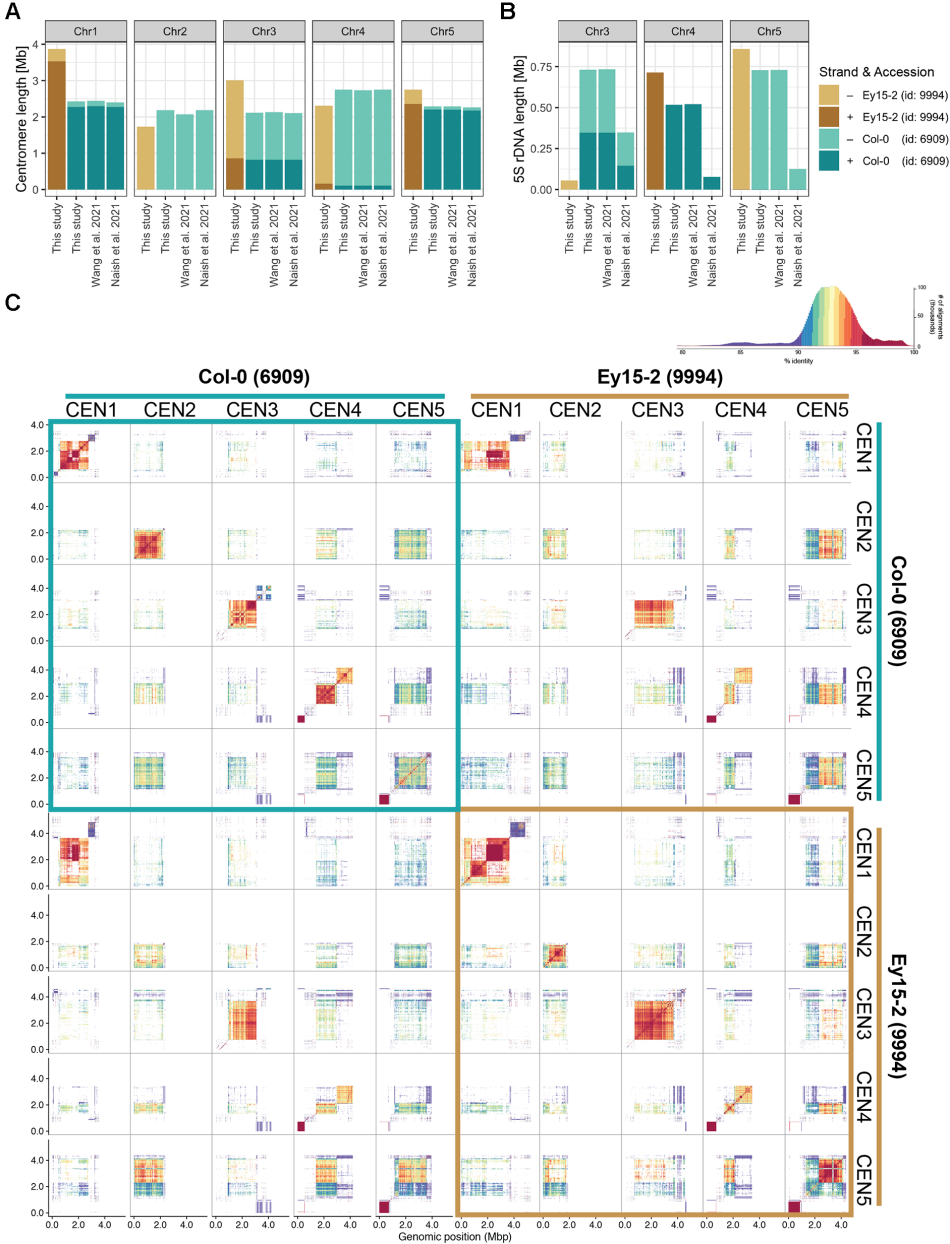
Centromere and 5S rDNA variation between *A. thaliana* accessions Ey15-2 and Col-0. (**A**) Centromere and (**B**) 5S rDNA length of each chromosome in the HiFi-Hifiasm assembly of accession Ey15-2 and three independent assemblies of accession Col-0: HiFi-Hifiasm in this study, ONT + HiFi in Wang *et al.* (66) and ONT + HiFi in Naish *et al.* (19). (**C**) Comparison of all pericentromeric regions in the HiFi-Hifiasm assemblies of Col-0 and Ey15-2 visualized by StainedGlass (71). A histogram of the colored percent identity is shown at the top-right of the panel.

Two recently published assemblies of the reference accession Col-0 have fully (19) or partially (66) resolved centromeres. Since our HiFi assemblies also provide access to previously unassembled regions of the nuclear genome (Figure 1C and 3B), most notably, centromeres, 5S rDNA clusters, and large insertions of organellar DNA, we compared these repetitive regions in our hybrid assembly of Ey15-2 with all existing assemblies of Col-0 (Figure 5A, B). Among the available Col-0 assemblies, there was high consistency in the length, orientation and overall structure for centromeres in chromosomes 1, 3, 4 and 5 (Figure 5A; Supplementary Figure S10). Only the centromere of chromosome 2 in the assembly from Wang *et al.* is slightly shorter, which could potentially be attributed to a gap in this assembly within the centromere (66).

In Col-0, CEN1 differs most from the other centromeres (19,66). Comparing our two accessions, CEN1 in Ey15-2 is at least 1.4 Mb longer than CEN1 in Col-0 (Figure 5A). Despite the length difference, CEN1 in Ey15-2 is more related to CEN1 in Col-0 than to any other Ey15-2 centromere (Figure 5C; Supplementary Figure S11a). In Ey15-2, there are two arrays encompassing CEN1, both larger than their counterpart in Col-0. The main array (upstream) consists of two distinct subarrays divided by a short inverted region (Supplementary Figure S11), and the downstream array is even more dissimilar to the other centromeres than the upstream one (Figure 5C). CEN2 is similar in size and orientation in Ey15-2 and Col-0, with Ey15-2 being ∼450 kb shorter (Figure 5A). CEN3 in Ey15-2 is ∼900 kb larger than in Col-0, the second largest size difference between homologous centromeres (Figure 5A). In spite of that, CEN3 of both accessions have the same inverted structure and they are also similar at the sequence level (Figure 5C). Although CEN4 is ∼440 kb shorter in Ey15-2 (Figure 5A), it has a similar bipartite structure as Col-0, with each array being very distinct (Figure 5C). As in Col-0, the upstream array is more similar to the other centromeres. The downstream array is more similar to its counterpart in Col-0 than to any other Ey15-2 centromere (Supplementary Figure S11b). Finally, CEN5 is >460 kb longer in Ey15-2 (Figure 5A), and it has fewer switches in strand orientation of satellite arrays (Supplementary Figure S11).

Regarding the 5S rDNA clusters, while their size and orientation were highly consistent between our Col-0 HiFi assembly and the one from Wang *et al.* (66) for chromosomes 3, 4 and 5, they were substantially smaller in the assembly from Naish *et al.* (19) for all three loci (Figure 5B). An important distinction between the two recently published Col-0 assemblies is that despite both being hybrid assemblies of ONT and Pacbio HiFi reads, one is primarily ONT-based (19) while the other is ultimately HiFi-based (66). The 5S rRNA gene copy number in Col-0 was previously estimated to be >2000 by quantitative PCR, which was considered an underestimate given that the primers may have missed units due to polymorphisms (56). With 1.98 Mb annotated as 5S rDNA, and considering that each 5S rDNA unit is ∼500 bp, our Col-0 HiFi assembly contains ∼3,962 5S rRNA genes while that of Naish *et al.* only ∼1,111 copies. Since the Col-0 individual we sequenced originated from the exact same seed batch as the one used for this previous study (19), and since 5S rRNA gene copy number has been shown to be rather stable in *A. thaliana* mutation accumulation lines propagated by single-seed descent (56), we speculate that this discrepancy likely reflects differences in the underlying long-read sequencing technologies (namely, PacBio HiFi versus ONT) and assembly algorithms, as opposed to a real biological difference between samples. To obtain a copy number estimate before the assembly process, we identified 5S rRNA genes directly on the q20 HiFi reads and, after normalizing by genome-wide read-depth, the estimate was 2983 copies. This is ∼1000 fewer copies than in the Col-0 HiFi assembly, but nearly 1900 more than in the assembly from Naish *et al.* While it remains challenging to determine the exact 5S rRNA gene number in the Col-0 genome, the latter estimate from unassembled long-reads is closer to both HiFi-based assemblies than to the ONT-based assembly.

When comparing Ey15-2 and Col-0 genomes, the orientation and size of the major 5S rDNA clusters in the upper arms of chromosomes 4 and 5 are similar, and only slightly larger in Ey15-2 (Figure 5B). Also, the minor 5S rDNA cluster on the lower arm of chromosome 5 is conserved (Supplementary Figure S12). In contrast, 5S rDNA repetitive elements make up only 55 kb of chromosome 3 in Ey15-2, that is, depending on whether we compared with the ONT-based or HiFi-based assemblies, six to thirteen times less than in Col-0. Presence/absence variation of 5S rDNA clusters in chromosome 3 between *A. thaliana* accessions is well known from cytological studies (56,77,78). With telomere-to-telomere assemblies that fully resolve centromeric and pericentromeric regions, we can now add several layers of resolution to these comparisons. Besides characterizing the actual length and orientation of the polymorphic 5S rDNA clusters themselves (Figure 5B), we can better appreciate their genomic neighborhood. For instance, from the two 5S rDNA clusters on the lower arm of chromosome 3 in Col-0 that are in different strand orientation, Ey15-2 only carries a minor version of the downstream cluster on the negative strand (Supplementary Figure S12).

As for organellar DNA insertions into the nuclear genome, the large mitochondrial DNA insertion near CEN2 in Col-0 is absent in Ey15-2 (Supplementary Figure S11). Although this insertion remains only partially characterized in the TAIR10 reference genome, fiber-fluorescence *in situ* hybridization analyses have shown it is ∼620 kb long (79). The large mitochondrial DNA insertion represents another locus inconsistent among the three Col-0 assemblies. While in the assembly from Naish *et al.* (19) it is 369 kb long, in our HiFi-Hifiasm Col-0 assembly and the one from Wang *et al.* (66) it is 640 kb long (Supplementary Figure S13), in remarkable agreement with the previous cytological estimate (79).

## DISCUSSION

Here, we have compared a CLR genome assembly that rivals the best published *A. thaliana* CLR assemblies with different HiFi assemblies produced with five state-of-the-art HiFi assemblers of the same sample. We find that a high-quality HiFi data set is preferable and, although a hybrid assembly of these two technologies accomplished a ‘telomere’-to-telomere genome (except for the two telomeres immediately adjacent to 45S rDNA arrays on chromosomes 2 and 4) with a single gap, only minor gains can be achieved by adding CLR data. An important insight is how much the choice of HiFi assemblers matters, to which we can confidently speak because we systematically compared their performance with the same long-read datasets. In *A. thaliana*, the HiFi assemblers FALCON, HiCanu and Hifiasm allowed us to access nearly 15 Mb more nuclear DNA sequence than the CLR assembly, primarily in the form of centromeres and 5S rDNA clusters (Figure 3A), with negligible differences in the non-repetitive fraction of the genome (Table 1). Hifiasm was our preferred choice because it achieved not only the highest consensus quality, but also because contiguity of the assembly was highly robust to a decrease in coverage and median read length (Figure 2).

Despite HiFi reads supporting the successful assembly of centromeric regions, the contig breaks along several chromosome arms—usually thought to be less challenging than highly repetitive centromeres—were initially puzzling (Figure 1C). Many contigs that did not end with telomeres or 45S rDNA repeats carried TEs at their edges, and several could at first not be explained (Figure 3B). PacBio CLR and ONT assemblies for the two HiFi genomes sequenced in this study helped us to shed light on the underlying cause for the vast majority of these breaks: GA/TC low-complexity repeats, which are poorly represented in the source HiFi reads (Figure 4C and D). Encouragingly, the confirmed sizes of gaps due to this class of repeats were relatively small, ranging from 290 to 2326 bp (Figure 4B). We therefore strongly favor the HiFi technology for routinely obtaining chromosome-level assemblies with gapless centromeres without the need of complementary chromosome scaffolding techniques such as optical or chromosome contacts maps.

Based on the success of centromere assemblies, we are excited by the prospect of analyzing centromeres and 5S rDNA clusters from multiple accessions, given the intriguing observations we have already made in a comparison between Ey15-2 and Col-0. For example, it will be of interest to learn whether relatively conserved structural features, such as the bipartite centromere array in chromosome 4, is common, or whether the downstream array, which presents low CENH3 occupancy in Col-0 (19), has diverged and been lost in other accessions. Similarly, it will be interesting to learn whether CEN1 stands apart in other accessions as well, or whether certain centromeres are more restricted in length variation. As for the 5S rDNA clusters, the full reconstruction of these loci in other accessions will enable the identification of cluster-specific polymorphisms that can serve as reporters of the expression status of each cluster, which could have implications on the 3D organization of chromatin within the nucleus.

## DATA AVAILABILITY

Raw data generated for this study such as PacBio CLR and HiFi reads, and Illumina PCR-free paired-end reads can be accessed in the European Nucleotide Archive (ENA; https://www.ebi.ac.uk/ena/browser/home) under project accession number PRJEB50694. The final genome assemblies of *Arabidopsis thaliana* accessions Ey15-2 and Col-0 are deposited under accession numbers GCA_946499665 and GCA_946499705, respectively. Custom scripts and small files to reproduce the analyses in this study can be found in the dedicated GitHub repository (https://doi.org/10.5281/zenodo.7313866). Larger files, such as the hard-masked version of TAIR10, the main genome assemblies, annotation files and Bionano optical maps can be found at https://doi.org/10.5281/zenodo.7326462.

## Supplementary Material

gkac1115_Supplemental_Files
