# Supplementary material for: Pushing the limits of HiFi assemblies reveals centromere diversity between two Arabidopsis thaliana genomes
Source: Nucleic Acids Res. 2022 Dec 1;50(21):12309–27. doi: 10.1093/nar/gkac1115 (PMC9757041; doi:10.1093/nar/gkac1115)
Supplement: gkac1115_Supplemental_Files [file gkac1115_supplemental_files.zip › REVISED_CLRvsHiFi_NAR_SuppFigures_CORRECTED.171122.pdf]

SUPPLEMENTARY FILES

Supplementary File 1. Excel file with three sheets: contigs and super-scaffolds manually curated from the hybrid assemblies of Ey15-2; size estimates and information of CLR patches and Bionano gaps in the HiFi-Hifiasm assembly of Ey15-2; size estimates and information of gaps in the HiFi-Hifiasm assembly of Col-0.

Supplementary File 2. Metrics of the HiFi-based assemblies based on subsets of data performed by Hifiasm, HiCanu, FALCON, Peregrine and IPA.

SUPPLEMENTARY FIGURES

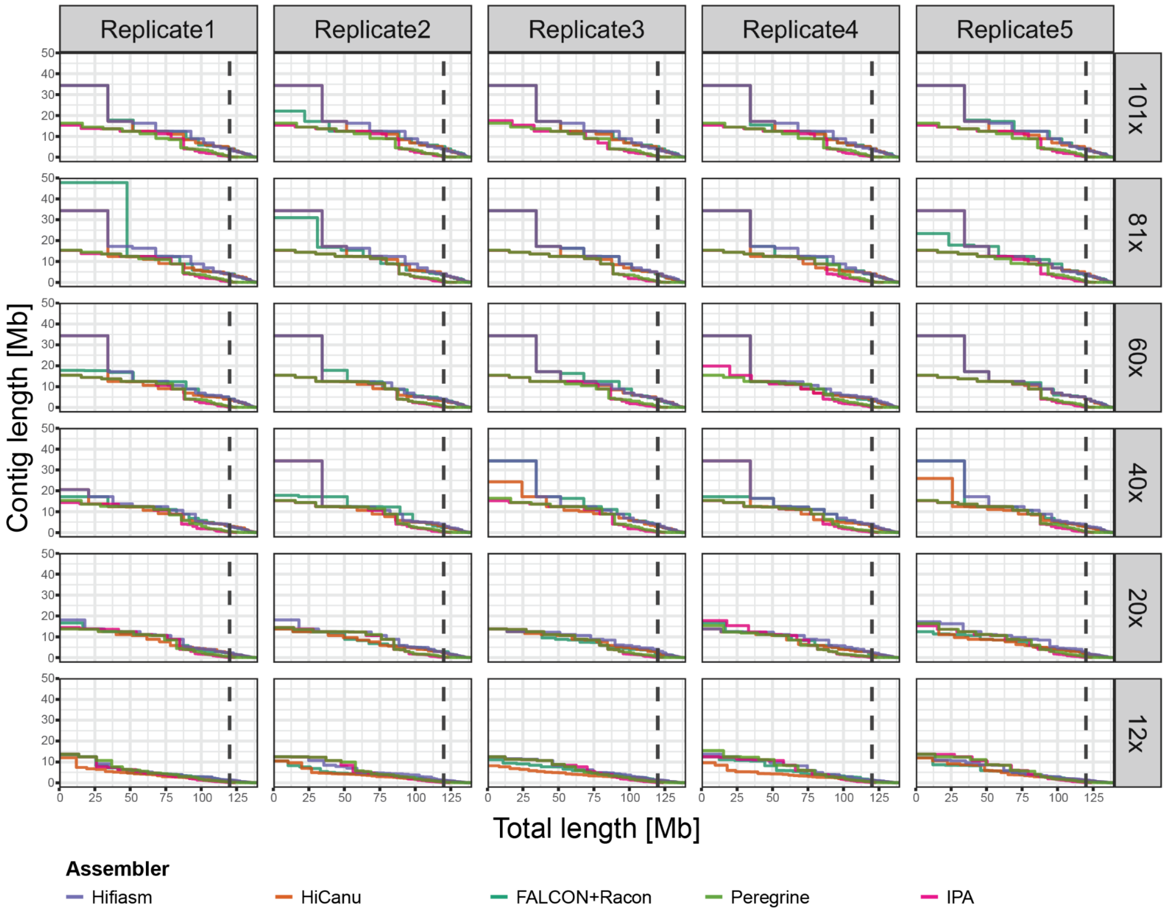

Supplementary Figure 1. The impact of coverage on five HiFi assemblers. Contiguity plots comparing 150 HiFi assemblies: five assemblers (indicated by different colors with key on the bottom; Hifiasm, HiCanu, FALCON, IPA, Peregrine), six coverage subsets (rows; 101x, 81x, 60x, 40x, 20x, 12x), with five replicates each (columns). For each assembly, the cumulative contig length (ordered from largest to shortest) is plotted over the estimated genome size of *A. thaliana* accession Ey15-2 (~143 Mb). The vertical dashed line indicates the size of the TAIR10 reference genome (119.14 Mb).

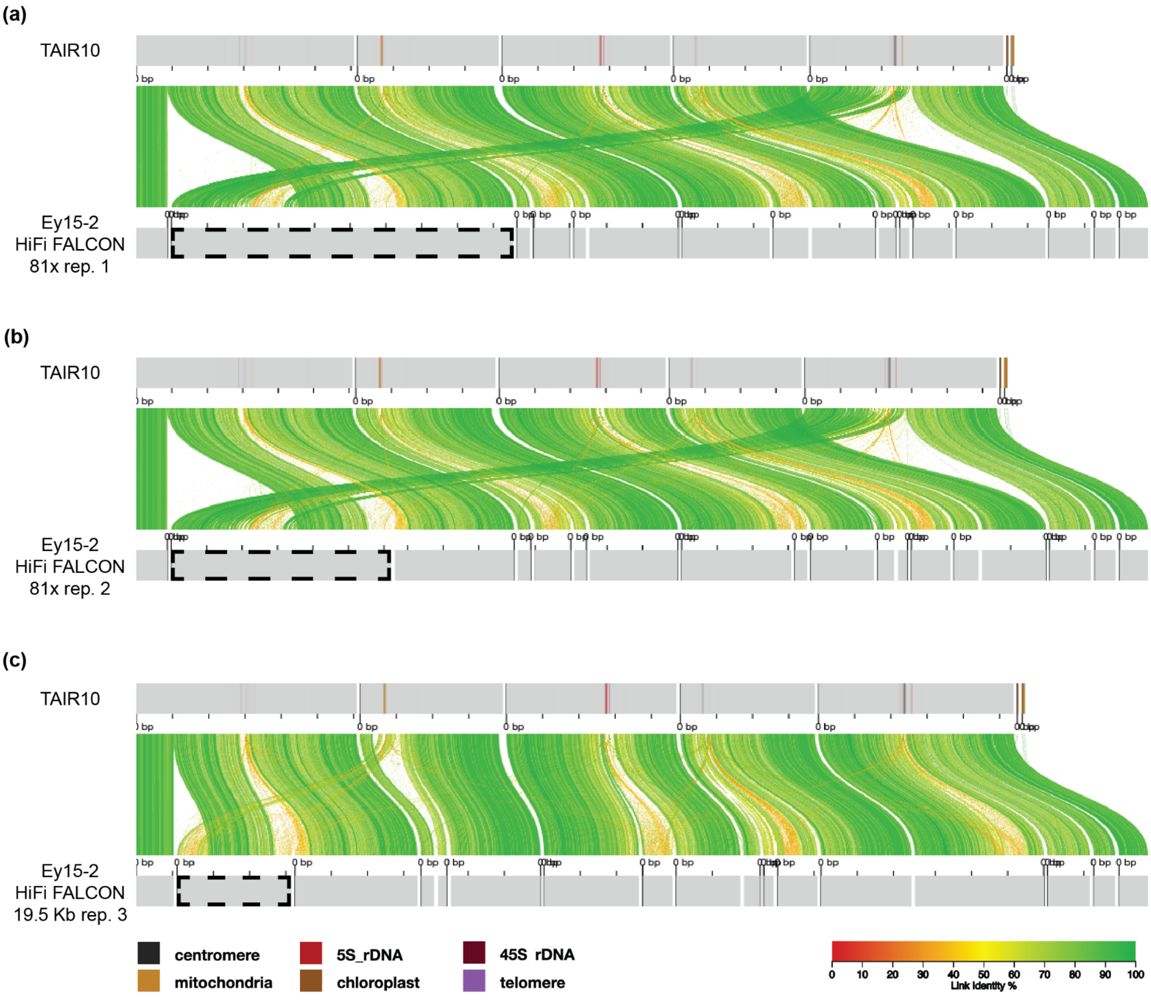

Supplementary Figure 2. Chimeric contigs produced by the HiFi assembler FALCON. Alignment of the TAIR10 reference genome and the contigs assembled by FALCON (24) with **(a)** coverage subset 81x replicate 1, **(b)** replicate 2, **(c)** and median read length subset 19.5 kb replicate 3 visualized by AliTV (70). Co-linear horizontal gray bars represent chromosomes or contigs, with sequence annotated as repetitive elements (centromeres, 5S and 45S rDNAs, telomeres, mitochondrial and chloroplast nuclear insertions) indicated by the colors shown on the bottom left. Only contigs >1 Mb are shown. Distance between ticks equals 10 Mb. Colored ribbons connect corresponding regions in the alignments.

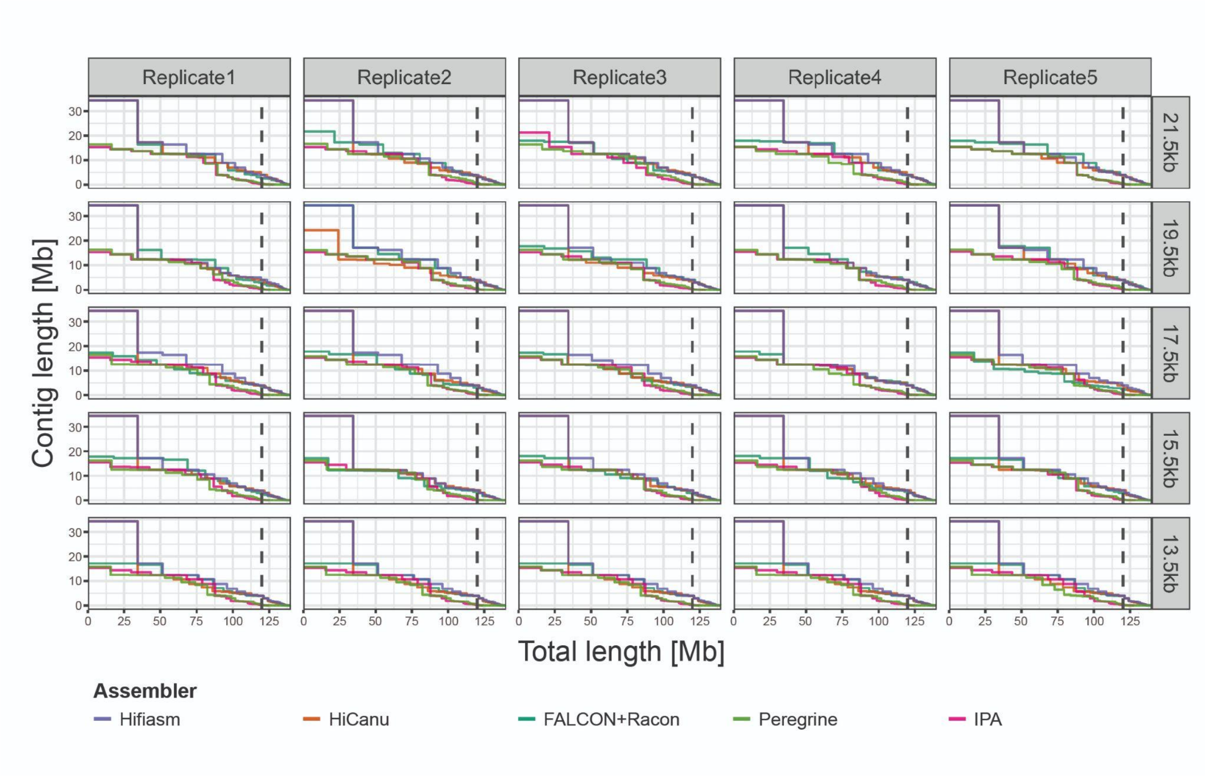

Supplementary Figure 3. The impact of read length on five HiFi assemblers. Contiguity plots displaying 125 HiFi assemblies: five assemblers (indicated by different colors with key on the bottom; Hifiasm, HiCanu, FALCON, IPA, Peregrine), five median read length subsets (rows; 21.5 kb, 19.5 kb, 17.5 kb, 15.5 kb, 13.5 kb), with five replicates each (columns). For each assembly, the cumulative contig length (ordered from largest to shortest) is plotted over the estimated genome size of *A. thaliana* accession Ey15-2 (~143 Mb). The vertical dashed line indicates the size of the TAIR10 reference genome (119.14 Mb).

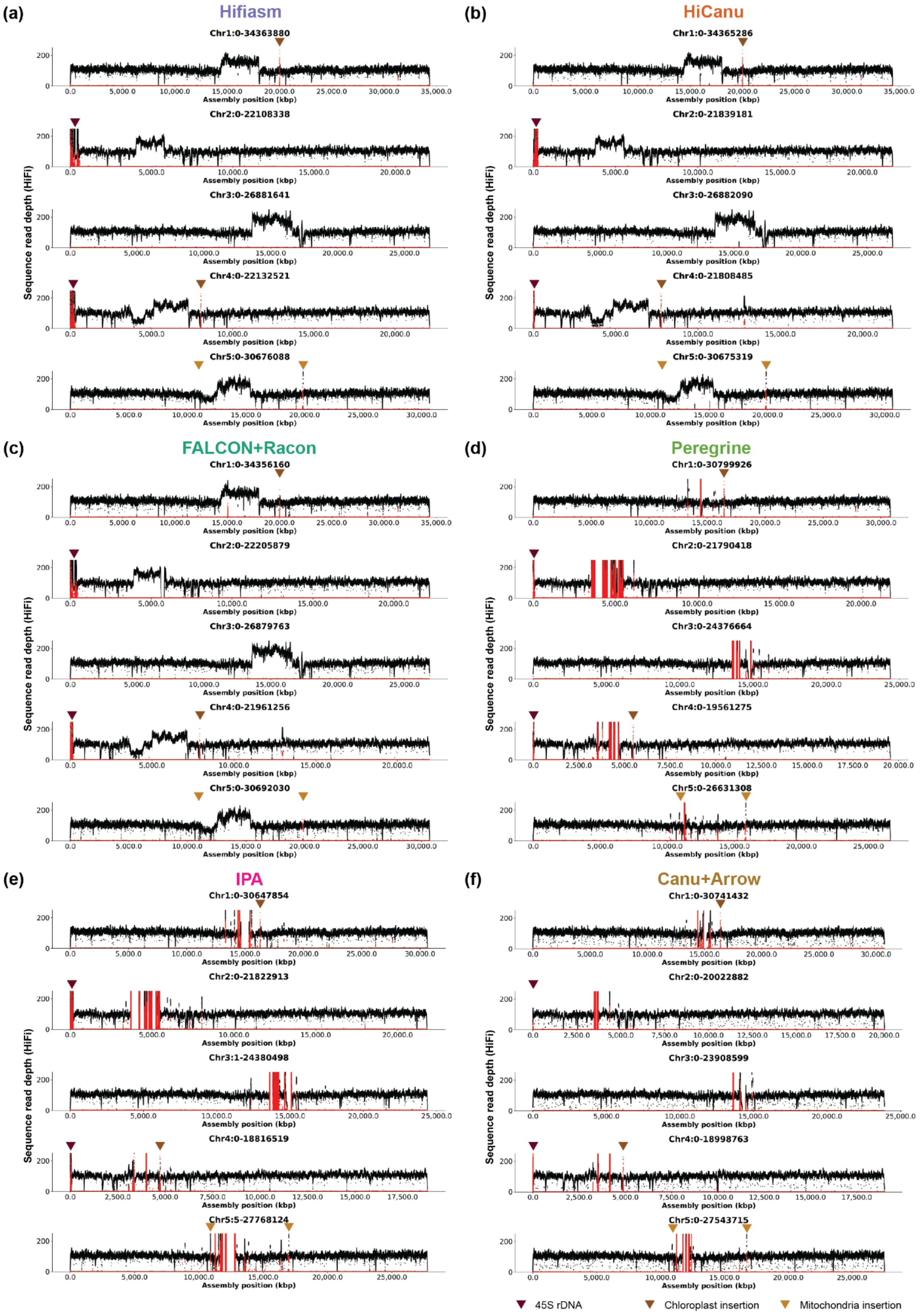

Supplementary Figure 4. Coverage of various Ey15-2 assemblies by HiFi read alignments. The coverage of primary (black) and secondary (red) alleles generated by NucFreq v0.1 (67) for HiFi reads aligned to **(a)** HiFi-Hifiasm, **(b)** HiFi-HiCanu, **(c)** HiFi-FALCON, **(d)** HiFi-Peregrine, **(e)** HiFi-IPA, and **(f)** CLR-Canu assemblies of Ey15-2. Since reads were aligned to nuclear chromosome scaffolds,

reads are expected to pile up at the top of chromosomes 2 and 4 corresponding to not fully scaffolded 45S rDNAs clusters, and at loci with organellar nuclear insertions. Therefore, 45S rDNA clusters, chloroplast and mitochondrial nuclear insertions >2 kb are indicated with inverted triangles by the colors shown on the bottom right.

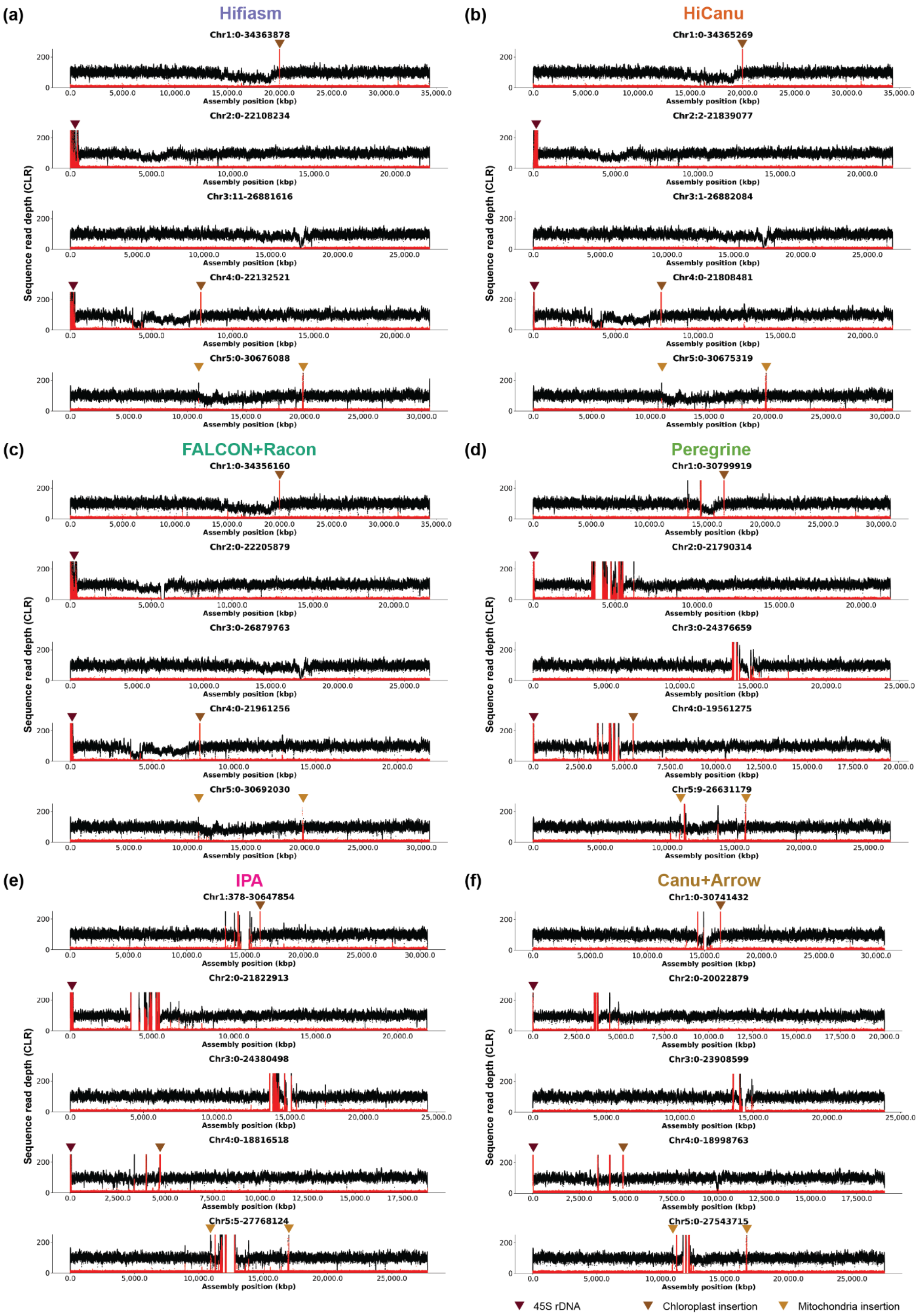

Supplementary Figure 5. Coverage of various Ey15-2 assemblies by CLRs. The coverage of primary (black) and secondary (red) alleles generated by NucFreq v0.1 (67) for CLRs aligned to **(a)** HiFi-Hifiasm, **(b)** HiFi-HiCanu, **(c)** HiFi-FALCON, **(d)** HiFi-Peregrine, **(e)** HiFi-IPA, and **(f)** CLR-Canu

assemblies of Ey15-2. Since reads were aligned to nuclear chromosome scaffolds, reads are expected to pile up at the top of chromosomes 2 and 4 corresponding to not fully scaffolded 45S rDNAs clusters, and at loci with organellar nuclear insertions. Therefore, 45S rDNA clusters, chloroplast and mitochondrial nuclear insertions >2 kb are indicated with inverted triangles by the colors shown on the bottom right.

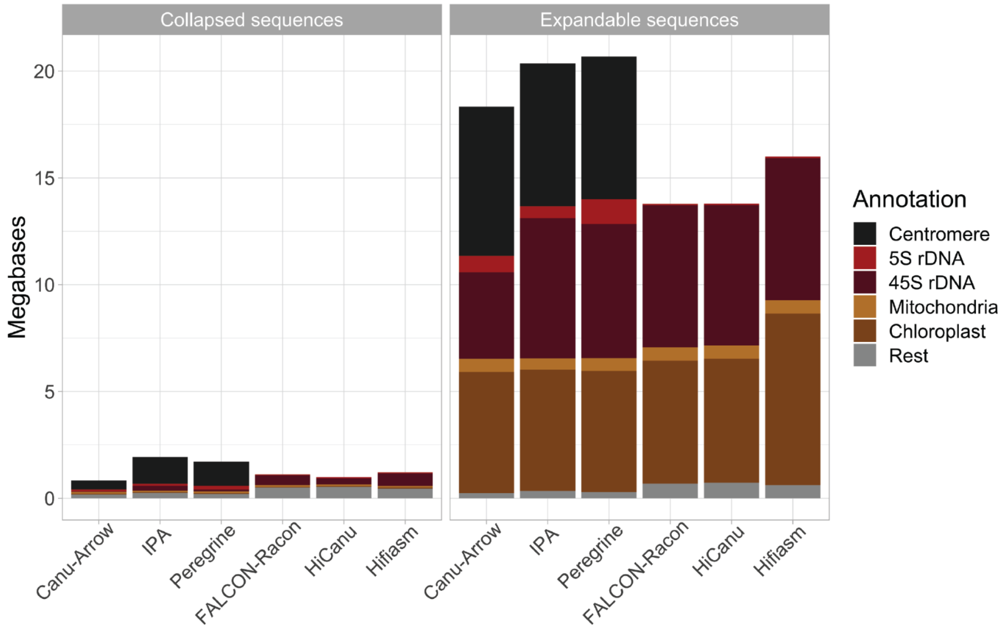

Supplementary Figure 6. Collapsed and expandable sequences in various Ey15-2 assemblies. Estimated amount of megabasepairs by SDA v0.1.0 (67) that are potentially collapsed (left) and that could be expanded (right) for the CLR-Canu and the five HiFi assemblies (Hifiasm, HiCanu, FALCON, IPA, Peregrine). The analysis is based on the distribution of CLR coverage along nuclear chromosome scaffolds, and collapsed/expandable sequences are partitioned according to their overlap with centromeres, rDNAs, or organellar nuclear insertions. Since reads are aligned to chromosome scaffolds, reads corresponding to not fully scaffolded 45S rDNAs clusters and organellar nuclear insertions are expected to disproportionately contribute to the fraction annotated as potentially expandable.

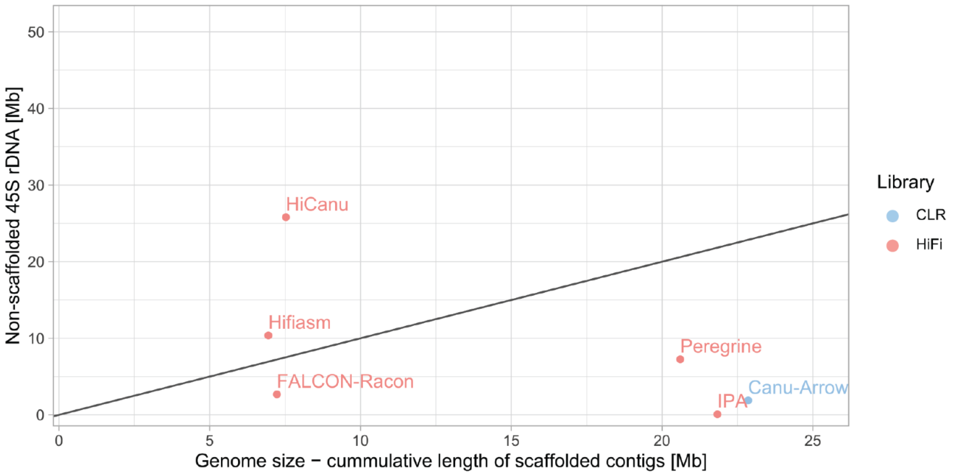

Supplementary Figure 7. Correlation between the missing portion of the genome and the non-scaffolded 45S rDNA sequence for various assemblers. The solid line indicates the one-to-one relationship between both axes.

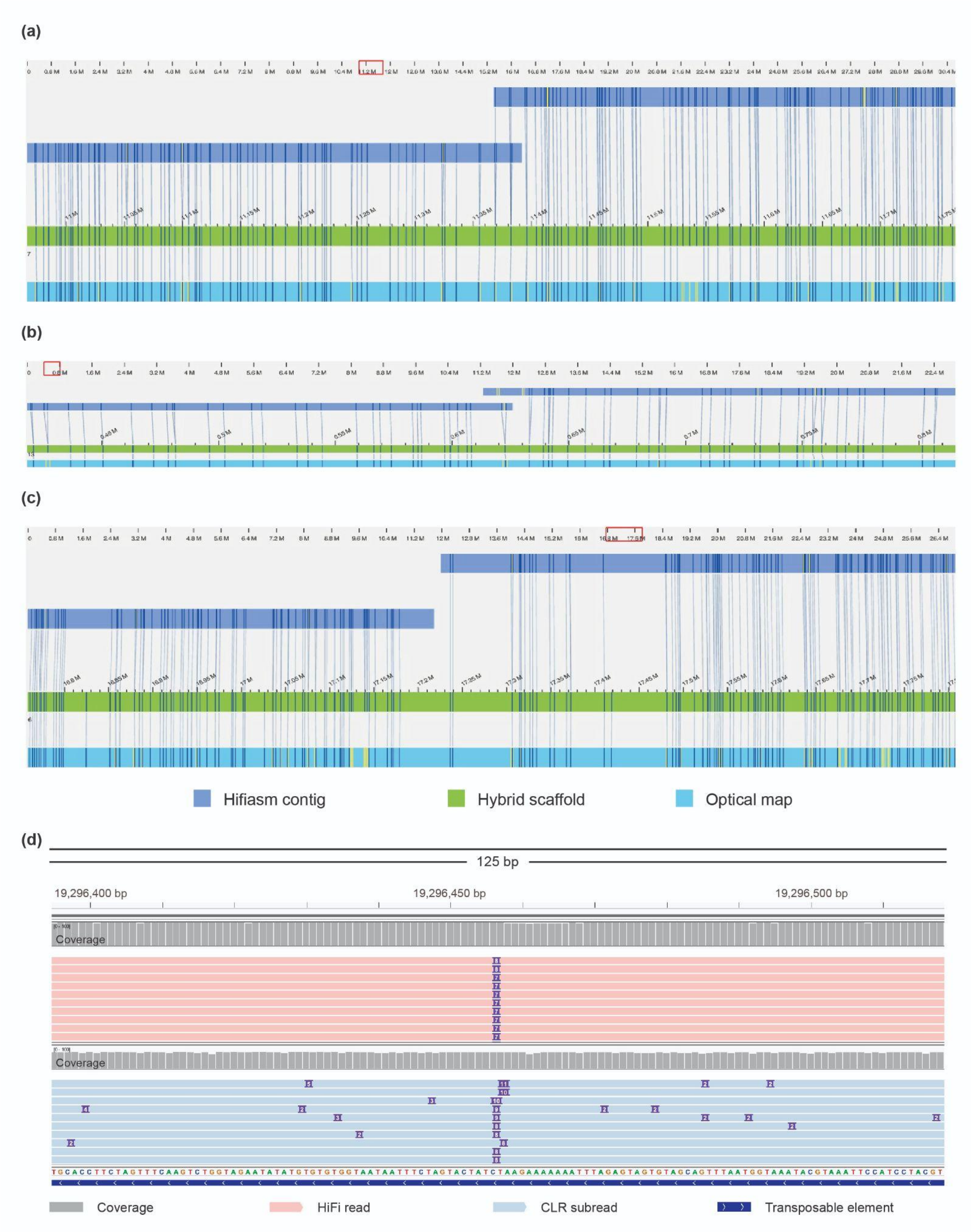

Supplementary Figure 8. Gaps and overlapping contigs resolved in hybrid scaffolds. **(a)** Hybrid scaffold (in green) consisting of Hifiasm contigs (in dark blue) and the Bionano optical map (in light blue) at an Ey15-2 chromosome 5 locus that the Hifiasm assembly alone was not able to resolve, producing a pair of overlapping contigs. (**b**) Similar to (a) for a chromosome 2 locus that reveals the inconsistency in labeling patterns for one of the contig edges. **(c)** Similar to (a) for a chromosome 3 locus where adjacent contigs did not overlap, creating a gap. Vertical lines connect consistent labeling

positions at DLE-1 recognition sites between long read contigs and Bionano optical maps. **(d)** Visualization with IGV (55) of aligned HiFi reads (in red; top) and CLRs (in blue; bottom) over Chr5:19296395-19296519 in the HiFi + CLR hybrid assembly. In the original HiFi-Hifiasm scaffold, the corresponding locus had a gap due to an unresolved contig overlap (a), but RagTag identified and fixed it, albeit leaving a 7 bp deletion, apparent from the alignment of long reads.

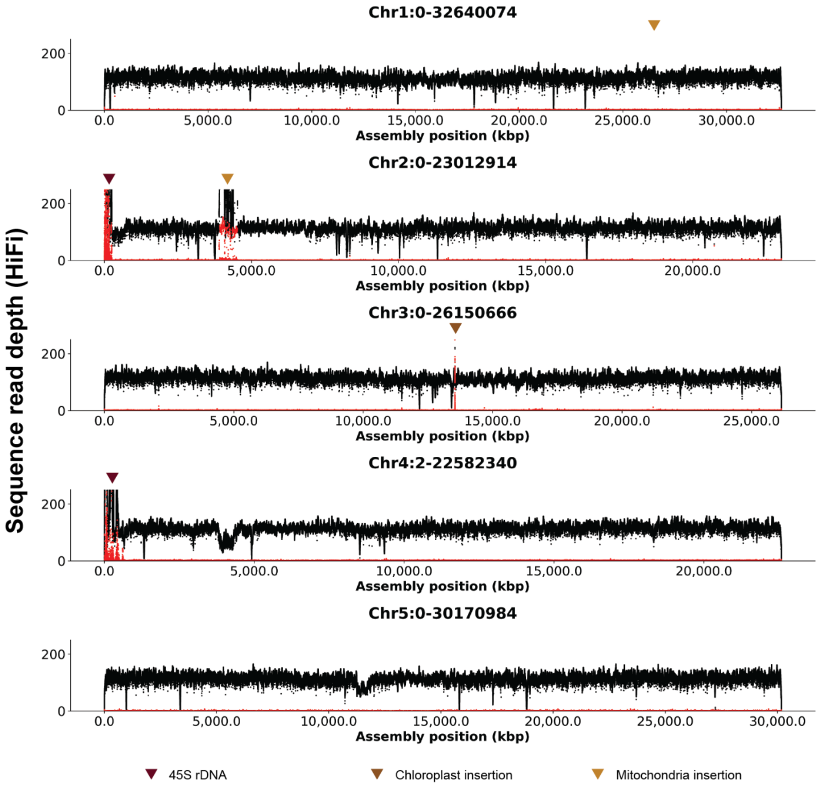

Supplementary Figure 9. The coverage of HiFi reads aligned to the Col-0 assembly. The coverage of primary (black) and secondary (red) alleles generated by NucFreq v0.1 (67) for HiFi reads aligned to the HiFi-Hifiasm assembly of Col-0. Since reads were aligned to nuclear chromosome scaffolds, reads are expected to pile up at the top of chromosomes 2 and 4 corresponding to not fully scaffolded 45S rDNAs clusters, and at loci with organellar nuclear insertions. Therefore, 45S rDNA clusters, chloroplast and mitochondrial nuclear insertions >2 kb are indicated with inverted triangles by the colors shown on the bottom.

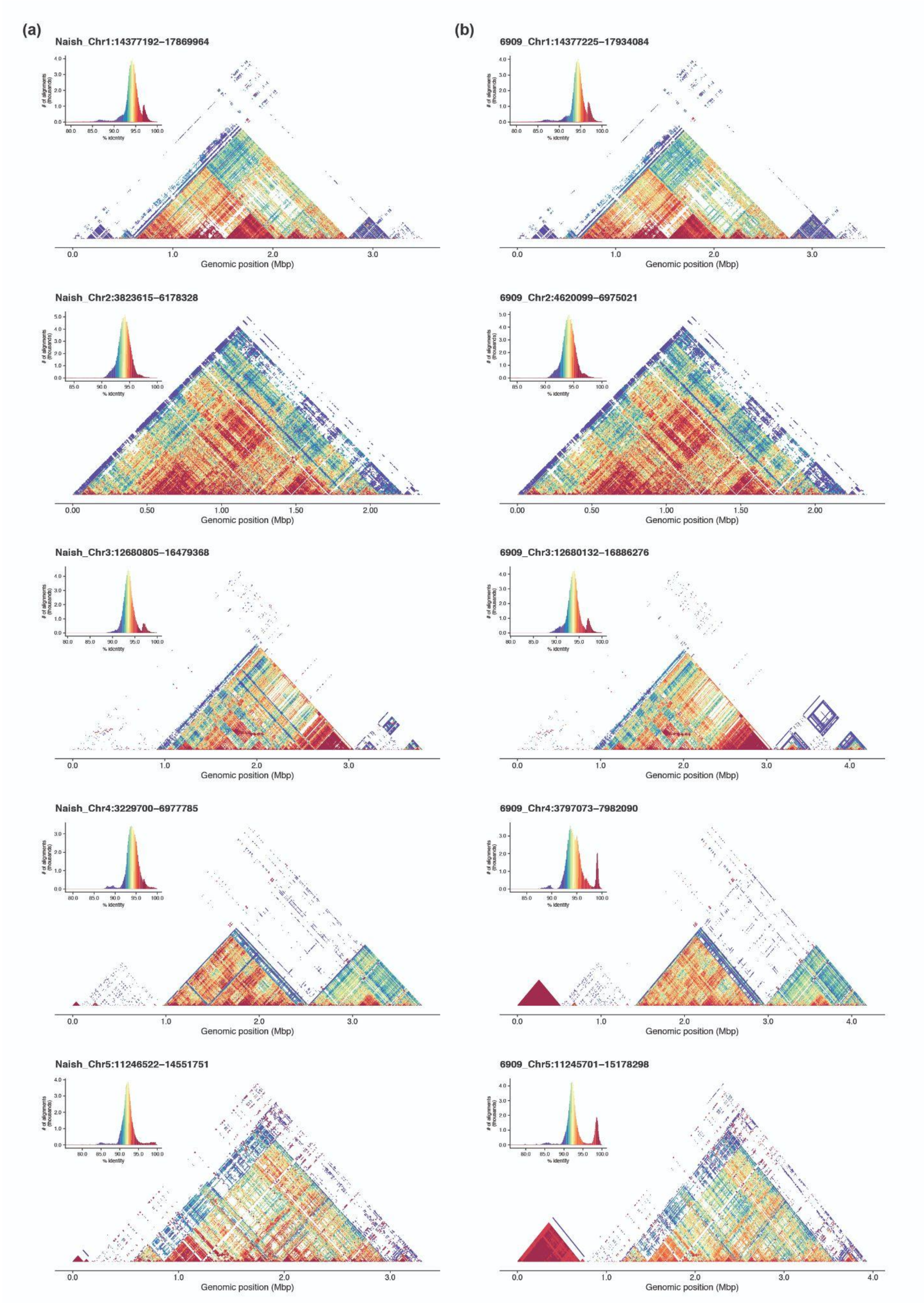

Supplementary Figure 10. StainedGlass (71) comparisons of pericentromeric regions in the **(a)** Naish *et al.* Col-0 assembly (19) and **(b)** the HiFi-Hifiasm Col-0 assembly produced here. While centromeres are largely consistent, 5S rDNA clusters are not. Histograms of the colored percent identity per centromere are also shown.

(a)

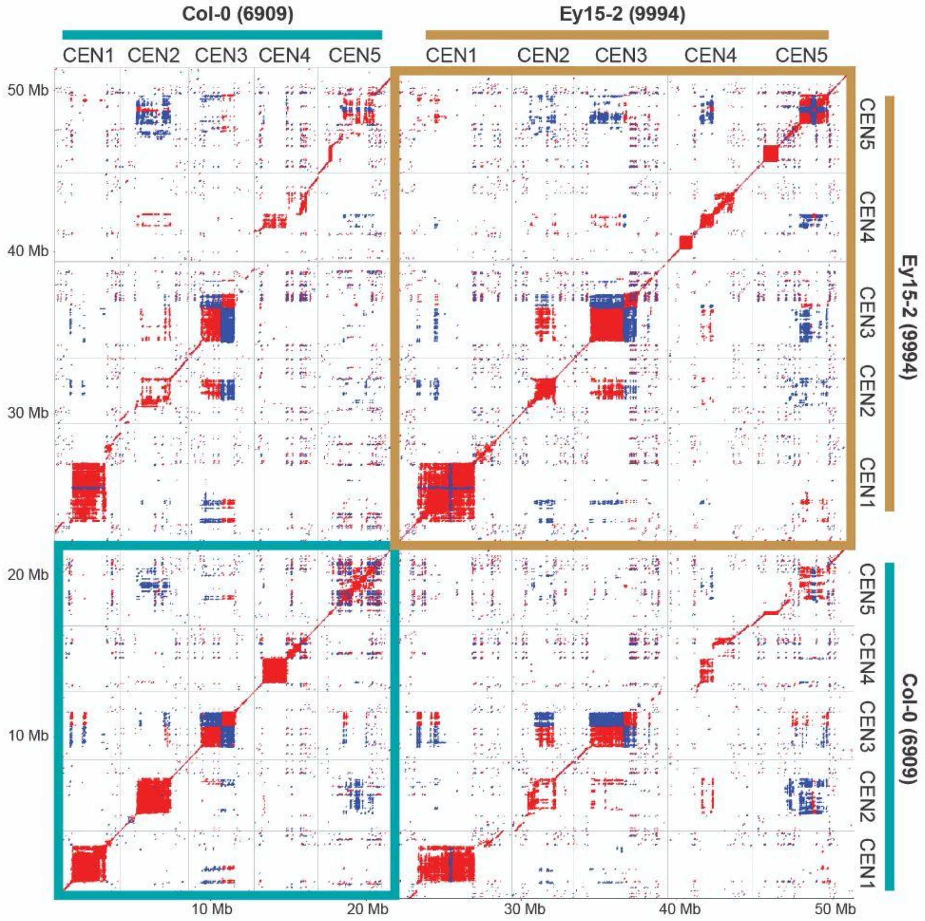
Col-0 (6909)
Ey15-2 (9994)
CEN1 CEN2 CEN3 CEN4 CEN5
CEN1 CEN2 CEN3 CEN4 CEN5
50 Mb
40 Mb
30 Mb
20 Mb
10 Mb
10 Mb
20 Mb
30 Mb
40 Mb
50 Mb
CEN5 CEN4 CEN3 CEN2 CEN1
Ey15-2 (9994)
CEN5 CEN4 CEN3 CEN2 CEN1
Col-0 (6909)

(b)

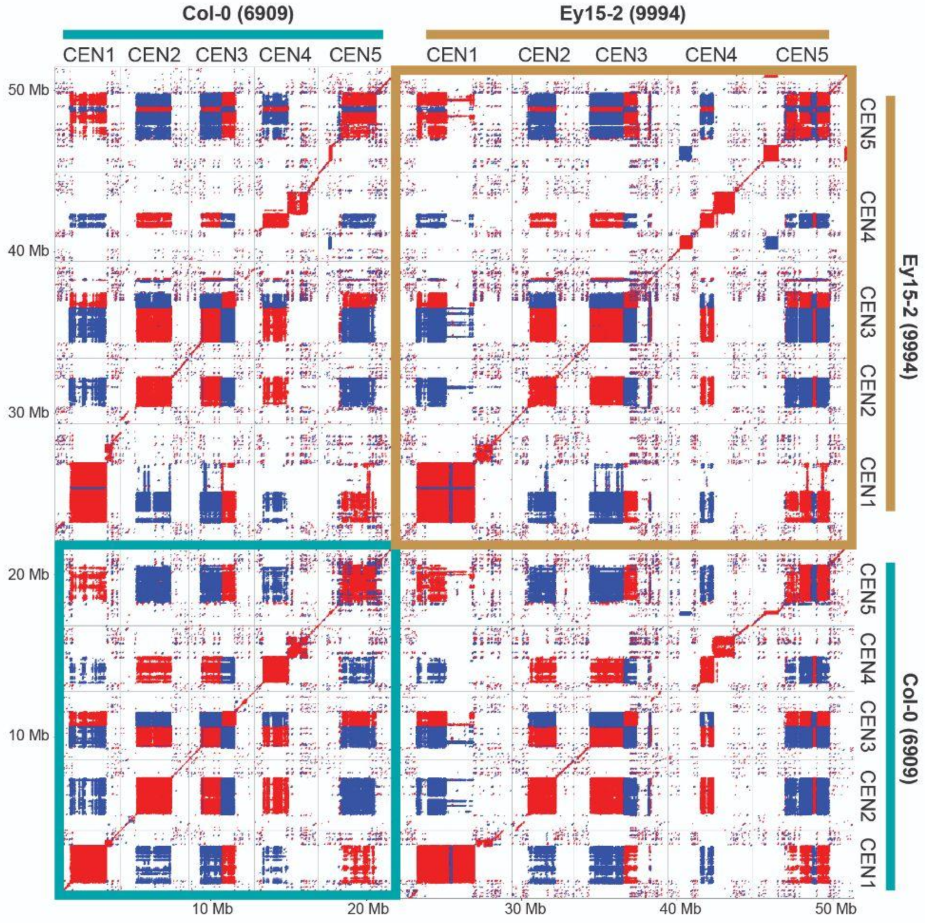
Col-0 (6909)
Ey15-2 (9994)
CEN1 CEN2 CEN3 CEN4 CEN5
CEN1 CEN2 CEN3 CEN4 CEN5
50 Mb
40 Mb
30 Mb
20 Mb
10 Mb
10 Mb
20 Mb
30 Mb
40 Mb
50 Mb
CEN5 CEN4 CEN3 CEN2 CEN1
Ey15-2 (9994)
CEN5 CEN4 CEN3 CEN2 CEN1
Col-0 (6909)

Supplementary Figure 11. Dotplot analysis comparing the five pericentromeric regions of Col-0 (19) and Ey15-2. **(a)** Using a search window of 178 bp. **(b)** Using a search window of 130 bp. Red and blue shading indicate detection of similarity on the same or opposite strands, respectively.

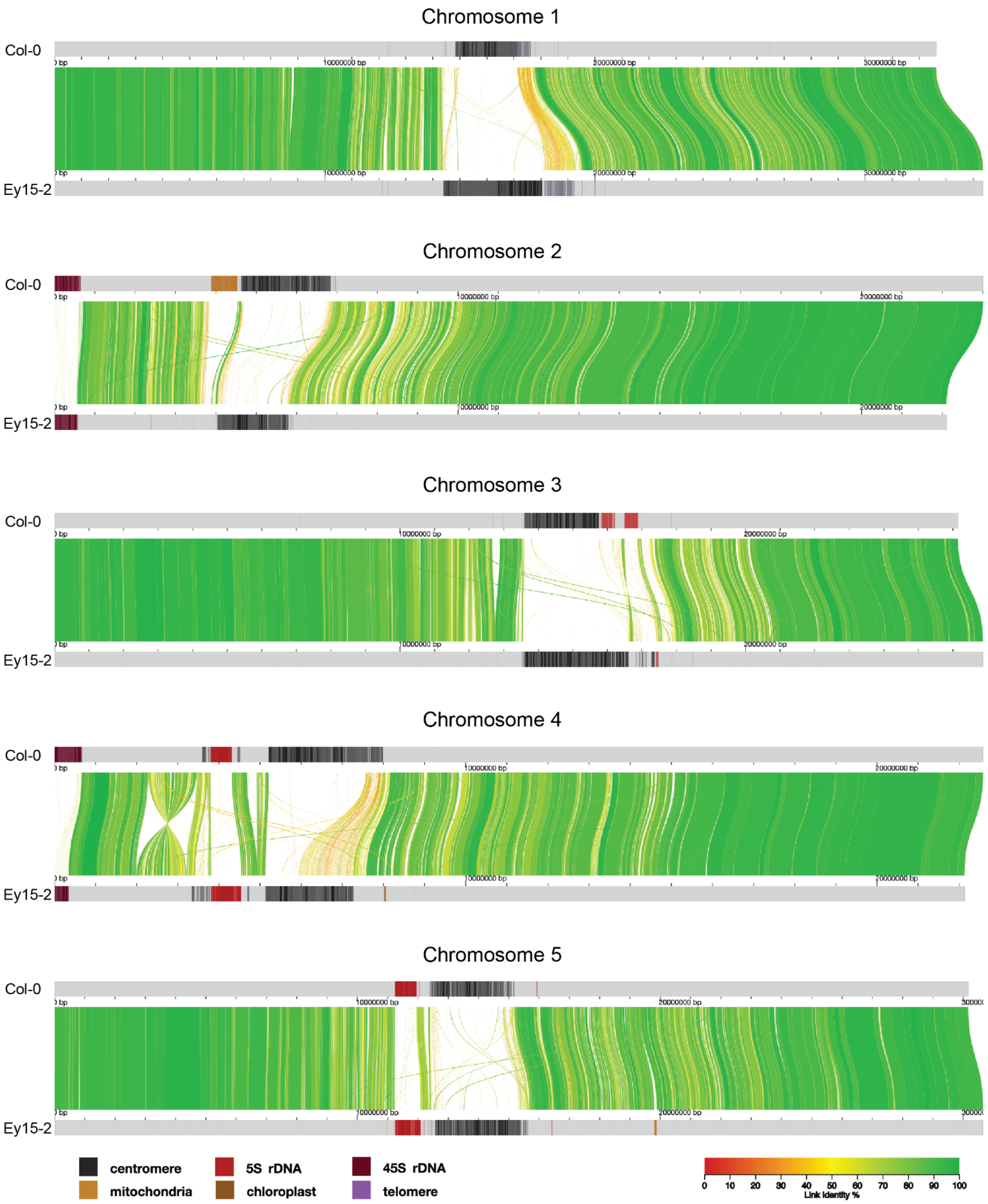

Supplementary Figure 12. Alignment of the HiFi + RagTag scaffolds of Col-0 and the HiFi + CLR hybrid assembly of Ey15-2 visualized by AliTV (70). Co-linear horizontal gray bars represent the five chromosomes in *A. thaliana*, with sequence annotated as repetitive elements (centromeres, 5S and 45S rDNAs, telomeres, mitochondrial and chloroplast nuclear insertions) indicated by the colors shown on the bottom left. Distance between ticks equals 1 Mb. Colored ribbons connect corresponding regions in the alignment.

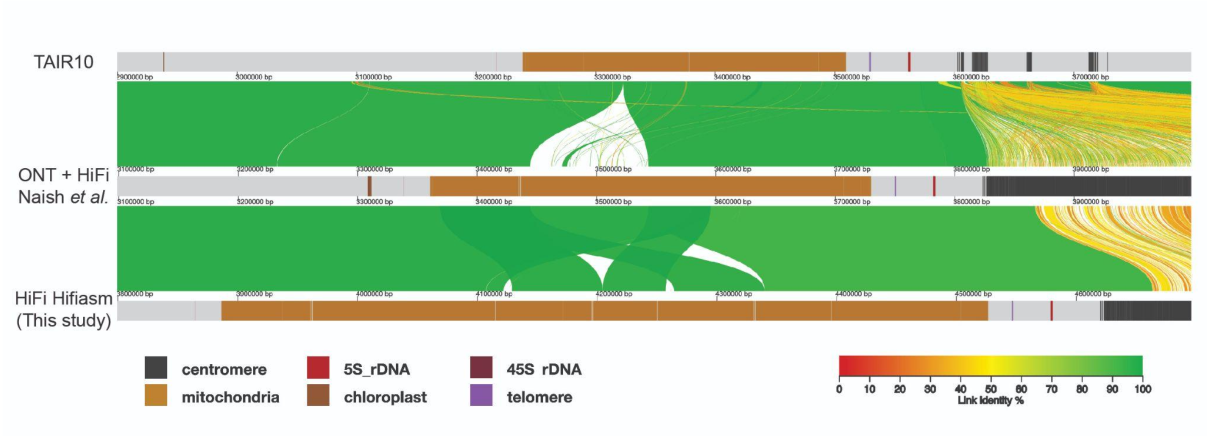

Supplementary Figure 13. Alignment of multiple assemblies of the mitochondrial DNA insertion near Col-0 CEN2 visualized by AliTV (70). Co-linear horizontal gray bars represent portions of the TAIR10 reference genome (*top*; (1)), the assembly from Naish *et al.* (*middle*; (19)), and the HiFi-Hifiasm Col-0 assembly produced here (*bottom*), with sequence annotated as repetitive elements (centromeres, 5S and 45S rDNAs, telomeres, mitochondrial and chloroplast nuclear insertions) indicated by the colors shown on the bottom left. Distance between ticks equals 100 kb. Colored ribbons connect corresponding regions in the alignment.
